# Supplementary material for: Dichography: two-frame ultrafast imaging from a single diffraction pattern
Source: Nat Commun. 2026 Jun 24;17:7944. doi: 10.1038/s41467-026-74442-w (PMC13448170; doi:10.1038/s41467-026-74442-w)
Supplement: Supplementary file 1 [file 41467_2026_74442_MOESM1_ESM.pdf]

Dichography: Two-frame Ultrafast Imaging from a Single Diffraction Pattern

Supplementary Information

S1 Brightness requirements of Dichography

S1.1 Simulation of realistic diffraction data

Similar to conventional CDI, the brightness of the diffraction image has a strong impact on the capability of *Dichography* to separate and retrieve the two frames contributing to the scattering. In this section, we empirically investigate this aspect by analyzing the performance of the method on simulated data.

Simulated diffraction images are produced following the procedure illustrated in Fig. S1. The inner part of the data is removed to mimic the presence of a hole in the detector, as required in real experiments to prevent damage from the transmitted FEL beam. The diameter of the missing central region is set to $10\,\%$ of the linear matrix size.

Statistical noise from photon counting is also added to the data. This noise depends on the overall signal brightness, i.e., the total photon count. The simulated noisy data, I^m, is generated using the following expression:

$$I^m_{ij} = \mathcal{P}\left[\frac{I^A_{ij}}{\sum I^A_{ij}} \cdot N^A_{\text{ph}}\right] + \mathcal{P}\left[\frac{I^B_{ij}}{\sum I^B_{ij}} \cdot N^B_{\text{ph}}\right] \tag{S1}$$

where $\mathcal{P}[x]$ denotes a Poisson-distributed random integer with mean x, and I^A and I^B are the simulated scattering intensities from ρ^A and ρ^B, respectively. The parameters N^A_{ph} and N^B_{ph} specify the desired total number of scattered photons for each contribution.

In this way, the simulated data I^m_{ij} represents photon counts for each pixel (i, j), with a total photon yield $\sum I^m_{ij}$ approximately equal to $N^A_{\text{ph}} + N^B_{\text{ph}}$, subject to statistical fluctuations.

For testing purposes, it is convenient to re-write the total photon counts of the two signals, N^A_{ph} and N^B_{ph}, as follows:

$$\begin{aligned} N^A_{\text{ph}} &= N^{\text{tot}}_{\text{ph}} \cdot \alpha \\ N^B_{\text{ph}} &= N^{\text{tot}}_{\text{ph}} \cdot (1-\alpha) \end{aligned} \tag{S2}$$

Here, $N^{\text{tot}}_{\text{ph}}$ indicates the total number of recorded photons after summing the two contributions, while $\alpha \in (0, 1)$ defines the balance between them. For example, $\alpha = 0.5$ means the contributions are perfectly balanced – so if $N^{\text{tot}}_{\text{ph}} = 2 \cdot 10^6$, then $N^A_{\text{ph}} = N^B_{\text{ph}} = 1 \cdot 10^6$. A value of $\alpha = 0.8$ instead means that $80\,\%$ of the scattered photons originate from ρ^A (i.e., $N^A_{\text{ph}} = 1.6 \cdot 10^6$) and $20\,\%$ from ρ^B (i.e., $N^B_{\text{ph}} = 4 \cdot 10^5$).

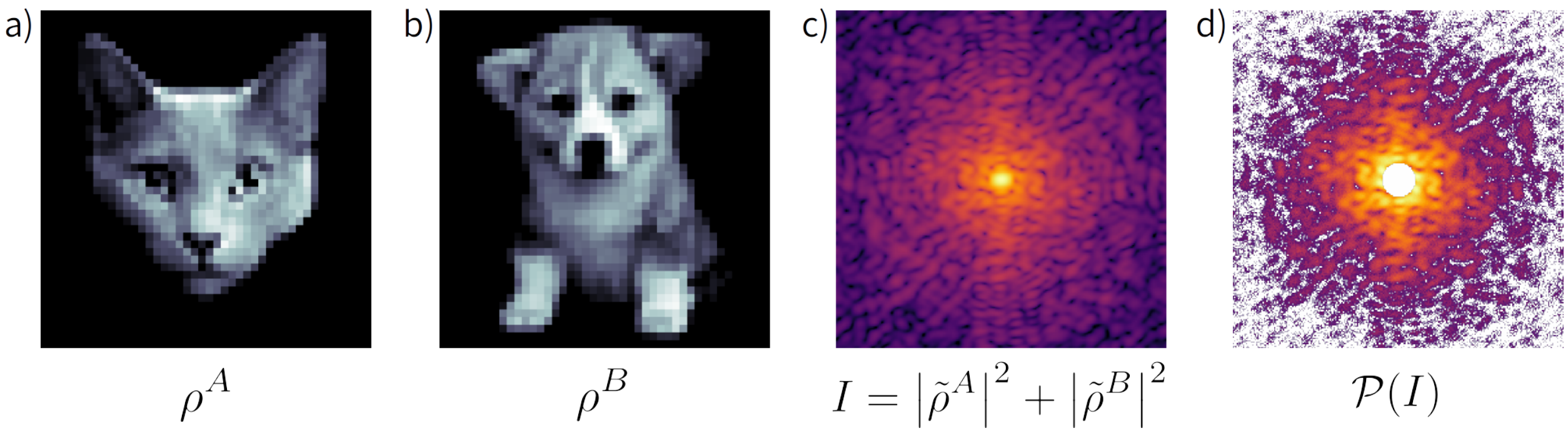

Figure S1: Example of simulated data. The simulations are performed by filling two real-valued matrices of size 266×256 pixels with two different images, as shown in a) and b). The scattered field is calculated as the incoherent sum of the two contributions, shown in c). Realistic data, shown in d), is produced by adding photon noise and removing the central part of the pattern, which is typically missing in FEL experiments to avoid detector damage from the transmitted beam. The images of the cat and the dog are adapted from [1] and [2], respectively.

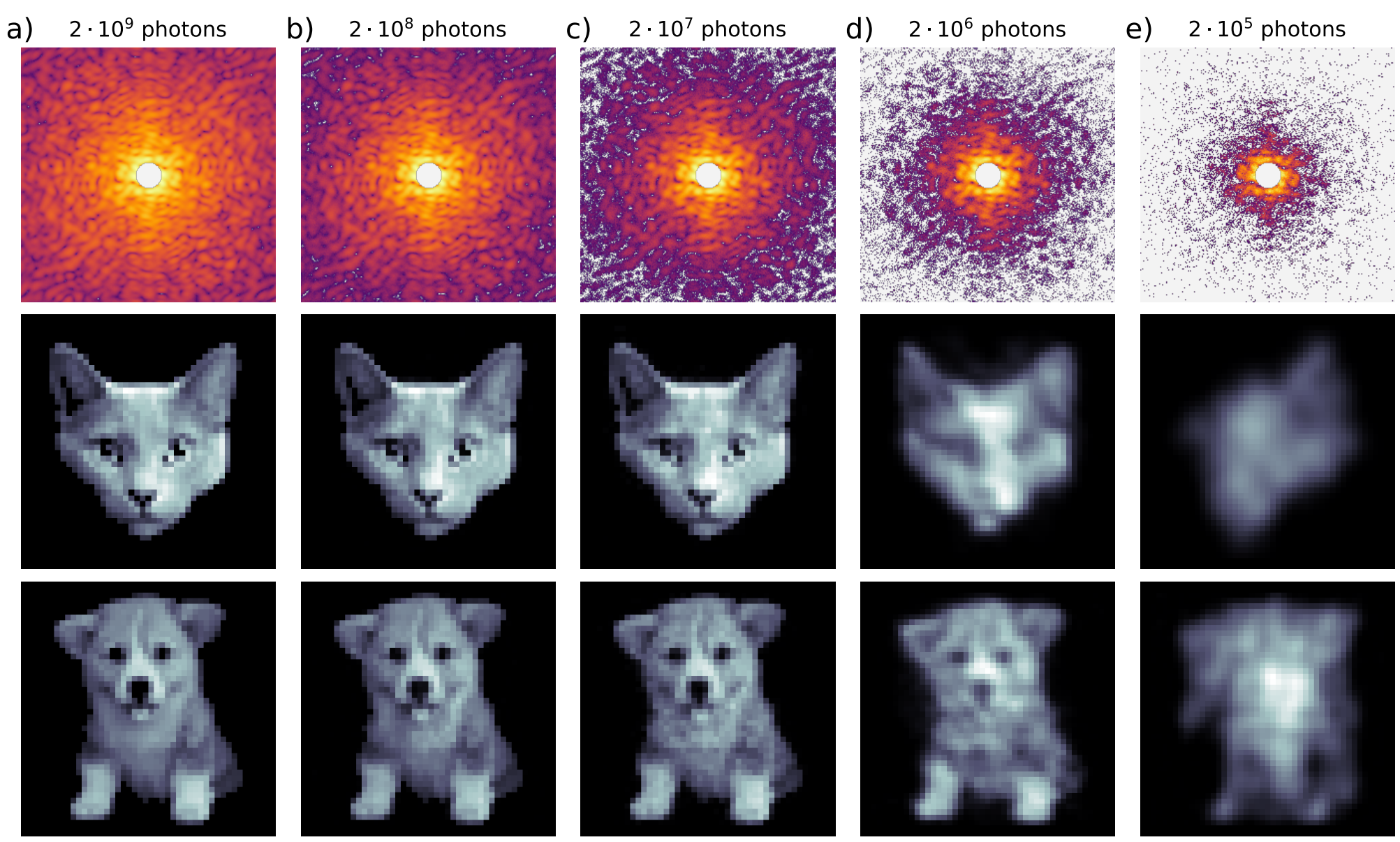

Figure S2: Impact of brightness on the reconstruction. Each subfigure a) to e) contains the diffraction image and the two reconstructed frames for different photon yields in the data.

In the following sections, the reconstruction capabilities of *Dichography* are tested for different total signal brightness values $N_{\text{ph}}^{\text{tot}}$ and varying balance α between the two scattering contributions. In those tests, the support function is supposed to be unknown, and it is retrieved in the reconstruction process.

S1.2 Effects of overall brightness

In this section, the results are tested for different values of the total photon count $N_{\text{ph}}^{\text{tot}}$, while keeping the individual contributions balanced at $50\,\%$ ($\alpha = 0.5$). Tests are performed on two different samples, and the corresponding results are presented in Fig. S2 and Fig. S3.

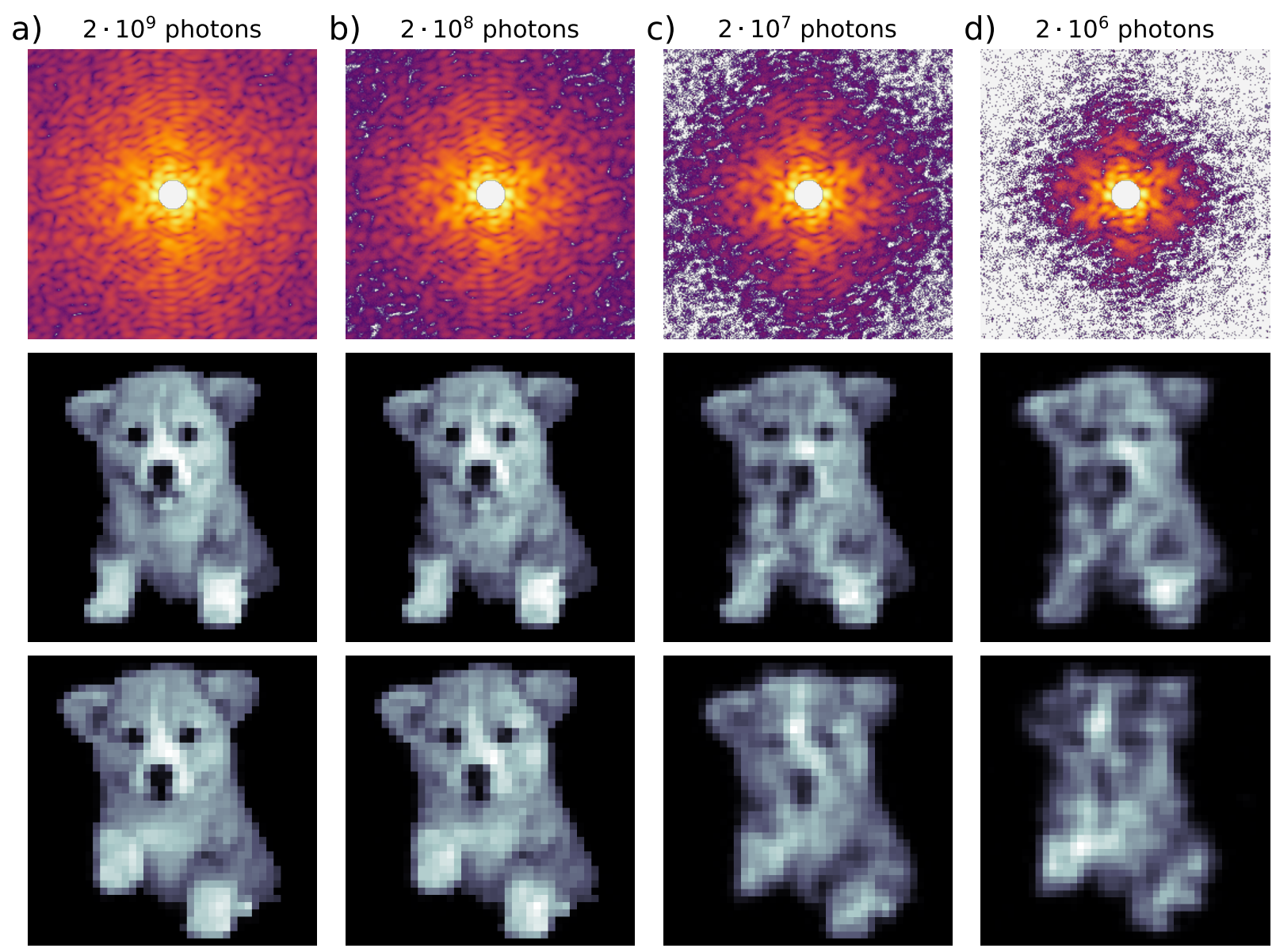

Figure S3: Impact of brightness on the reconstruction. Each subfigure a) to d) contains the diffraction image and the two reconstructed frames for different photon yields in the data.

The data in Fig. S2 originate from two densities whose spatial extensions (i.e., support functions) do not fully overlap and have significantly different shapes. In this case, the two frames are accurately retrieved down to $2 \cdot 10^7$ photons (Fig. S2c). Separation is still possible at $2 \cdot 10^6$ photons, although with a noticeable loss in spatial resolution (Fig. S2d). The reconstruction fails entirely at a photon yield of $2 \cdot 10^5$.

The problem becomes more brightness-sensitive in cases where the two samples have similar shapes, and the support function of one frame can fully contain the other. This situation is investigated in Fig. S3, and it is conceptually similar to the two-color case, where the sample is imaged with two different photon energies. The data for this example are produced by taking two similar images (the dog's mouth is open in the first frame, the paw is raised in the second) and scaling one to a size that differs by $10\,\%$ in pixel count. This mimics scattering from two photon energies with a $10\,\%$ difference in wavelength, making the second frame (corresponding to the longer wavelength) smaller in spatial extension.

Fig. S3 reveals a different behavior compared to Fig. S2. On the one hand, the ability to distinguish the two overall shapes persists down to $2 \cdot 10^6$ photons (Fig. S3d). On the other hand, *Dichography* struggles more to resolve fine details, with reconstruction artifacts already dominating at $2 \cdot 10^7$ photons (Fig. S3c).

S1.3 Relative intensities of the two frames

The second aspect investigated here concerns the relative intensity between the two frames, tuned via the parameter α in Eq. (S2).

Values of $\alpha = 0.5$, 0.7, 0.8, 0.9, and 0.95 are probed, corresponding to $50\,\%$-$50\,\%$, $70\,\%$-$30\,\%$, $80\,\%$-$20\,\%$, $90\,\%$-$10\,\%$, and $95\,\%$-$5\,\%$ photon distributions between ρ^A and ρ^B.

The first test, shown in Fig. S4, is performed on data with high brightness, with a total photon count of $N_{\text{ph}}^{\text{tot}} = 2 \cdot 10^9$. In the figure, the reconstruction of the second frame (dog) is shown twice: once normalized on an independent color scale, and once using the same scale as the first

frame. This highlights differences in scattering intensity and the resulting density contrast. In this high-brightness case, all reconstructions were successful, even for the highly unbalanced case in Fig. S4e, where the brightness of the first frame is 19 times higher.

The same photon balance configurations are tested at decreasing total photon counts: $N_{\text{ph}}^{\text{tot}} = 2 \cdot 10^8$, $2 \cdot 10^7$, and $2 \cdot 10^6$, shown in Fig. S5, Fig. S6, and Fig. S7, respectively.

From comparing these figures, it can be deduced that decreasing photon yield not only impacts reconstruction quality (as discussed in Fig. S2 and Fig. S3), but also makes *Dichography* increasingly sensitive to unbalanced contributions between the two signals. This effect can be explained and understood through statistical considerations of shot noise.

The primary source of noise in the data arises from the statistical nature of photon detection. As described in Eq. (S1), photon statistics follow a Poisson distribution. This implies that for a scattering signal with an expected value I_{ij} (expressed in photon counts), the standard deviation, i.e., the statistical fluctuation, is $\sqrt{I_{ij}}$.

Since the recorded signal is the sum of two independent Poisson-distributed random variables, the total measured intensity has an expected value of $I_{ij}^A + I_{ij}^B$ and a standard deviation of $\sqrt{I_{ij}^A + I_{ij}^B}$. Assuming that the first frame of the reconstruction is perfectly retrieved, i.e., I_{ij}^A is known exactly, the remaining signal attributed to the second frame can be defined as:

$$I_{ij}^{B_m} = \mathcal{P}\left[I_{ij}^A + I_{ij}^B\right] - I_{ij}^A \tag{S3}$$

with statistical properties:

$$\begin{aligned} \mu_{ij}^{B_m} &= I_{ij}^B = (1-\alpha) \cdot I_{ij} \\ \sigma_{ij}^{B_m} &= \sqrt{I_{ij}^A + I_{ij}^B} = \sqrt{I_{ij}} \end{aligned} \tag{S4}$$

The ability to extract meaningful information from $I_{ij}^{B_m}$ depends on achieving a sufficient Signal-to-Noise Ratio (SNR). To resolve the signal reliably, the SNR must exceed a minimum threshold $\mathcal{S}$:

$$\text{SNR}_{ij}^{B_m} = \frac{\mu_{ij}^{B_m}}{\sigma_{ij}^{B_m}} = (1-\alpha) \cdot \sqrt{I_{ij}} > \mathcal{S} \tag{S5}$$

This leads to a condition on the balance parameter α:

$$\alpha < 1 - \frac{\mathcal{S}}{\sqrt{I_{ij}}} \tag{S6}$$

The condition presented in Eq. (S6) is thus a simplified view of the system and cannot be interpreted in a fully quantitative manner. Still, it captures the qualitative behavior observed in Figures S4, S5, S6, and S7. Specifically, given a minimum SNR value $\mathcal{S}$ necessary for successful reconstruction, the higher the brightness of the pattern $I_{ij} \propto N_{\text{ph}}^{\text{tot}}$, the closer α can be to 1 (i.e., a strong imbalance between the signals), as shown in Fig. S4. Conversely, the lower the overall pattern intensity, the smaller the allowed values of α, as illustrated, for example, in Fig. S7.

The considerations made so far concern specific pixel coordinates, and low SNR values may have different impact on a CDI reconstruction. For example, if low SNR values are at high momentum transfer (close to the edges of the detector), the reconstruction outcome is typically successful and only the spatial resolution is affected. When low SNR values are already appearing at low momentum transfer, close to the center of the detector, the CDI reconstruction typically completely fails.

Furthermore, the amount of noise to which the reconstruction is tolerant is dependent on the

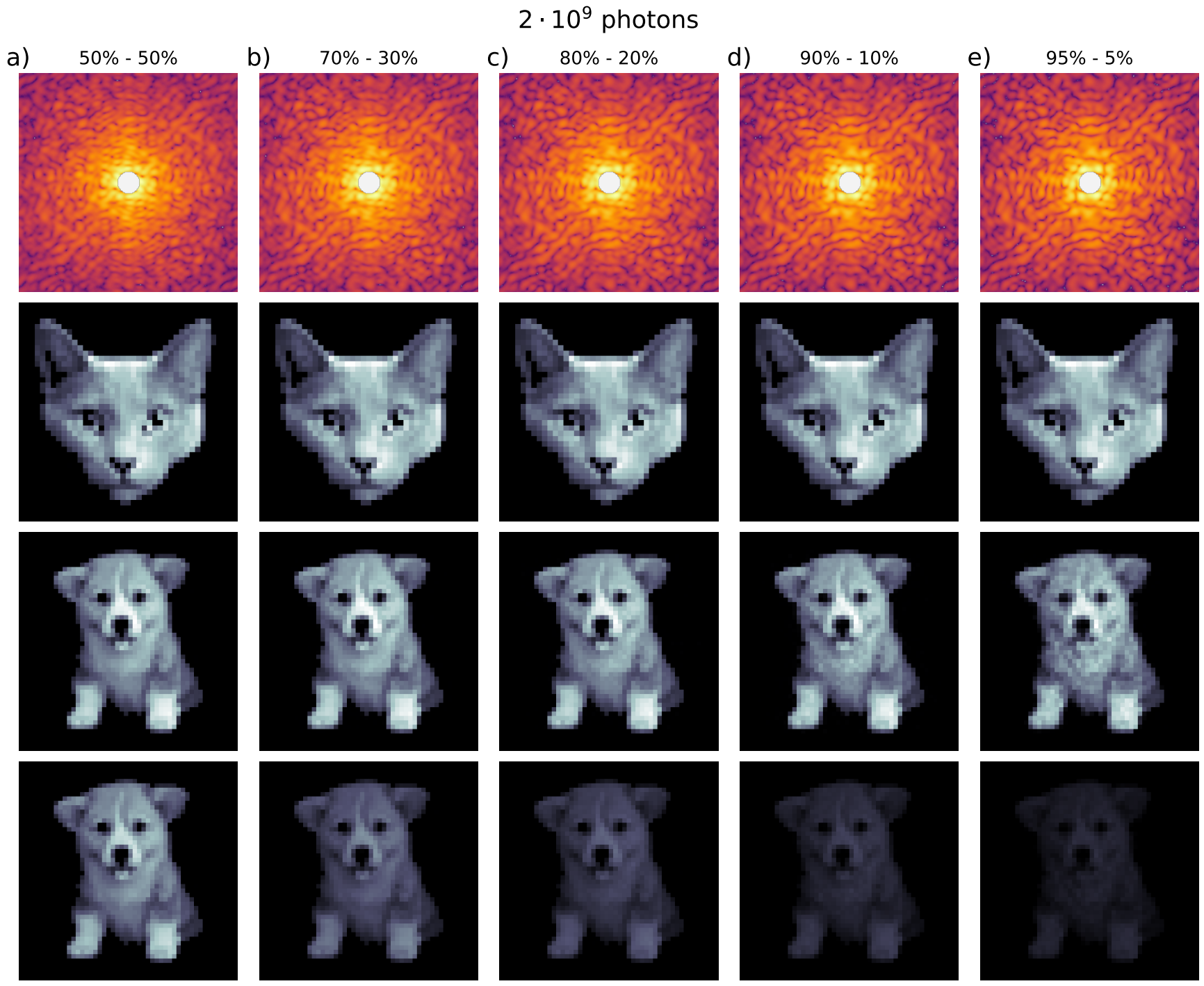

Figure S4: Impact of different relative brightness on the reconstruction for a total photon count of $2 \cdot 10^9$. Each subfigure a) to e) contains the diffraction image and the two reconstructed frames for different photon yields in the data. The second frame of the reconstruction is reported twice, in the third row with renormalized brightness, while in the fourth with the same color scale of the first frame.

sample, and in particular its size. At similar conditions in terms of brightness of the pattern, i.e., number of recorded photons, higher *oversampling degrees* (i.e., smaller samples) are typically much more resilient to noise than more extended samples, due to the lower number of unknowns to retrieve. Conversely, this observation is reversed when considering similar intensities in the focal region, which is a more realistic constraint. Indeed, the size of the support function (which defines the oversampling degree) corresponds to the geometrical cross-section of the sample and scales with the square of the sample dimension. The scattering cross section, at least for short wavelengths, increase instead with the volume of the sample. The ratio between recorded information and information to retrieve is thus favorable for larger samples.

Still, FEL diffraction data is always affected by the lack of scattering information in the central part of the detector, due to the presence of the main FEL beam. This, in practice, renders the reconstructions of larger samples more challenging and prone to artifacts. The lack of information in the center is, indeed, the actual limiting factor towards the reconstruction of larger samples. To summarize, the best conditions for imaging, and especially for Dichography for which the total intensity is crucial, are not trivial to identify and require specifically tailored diffraction simulations that include the FEL intensity, the color ratio, the scattering cross section of the sample material, the sample shape and size and the geometrical properties of the scattering detector.

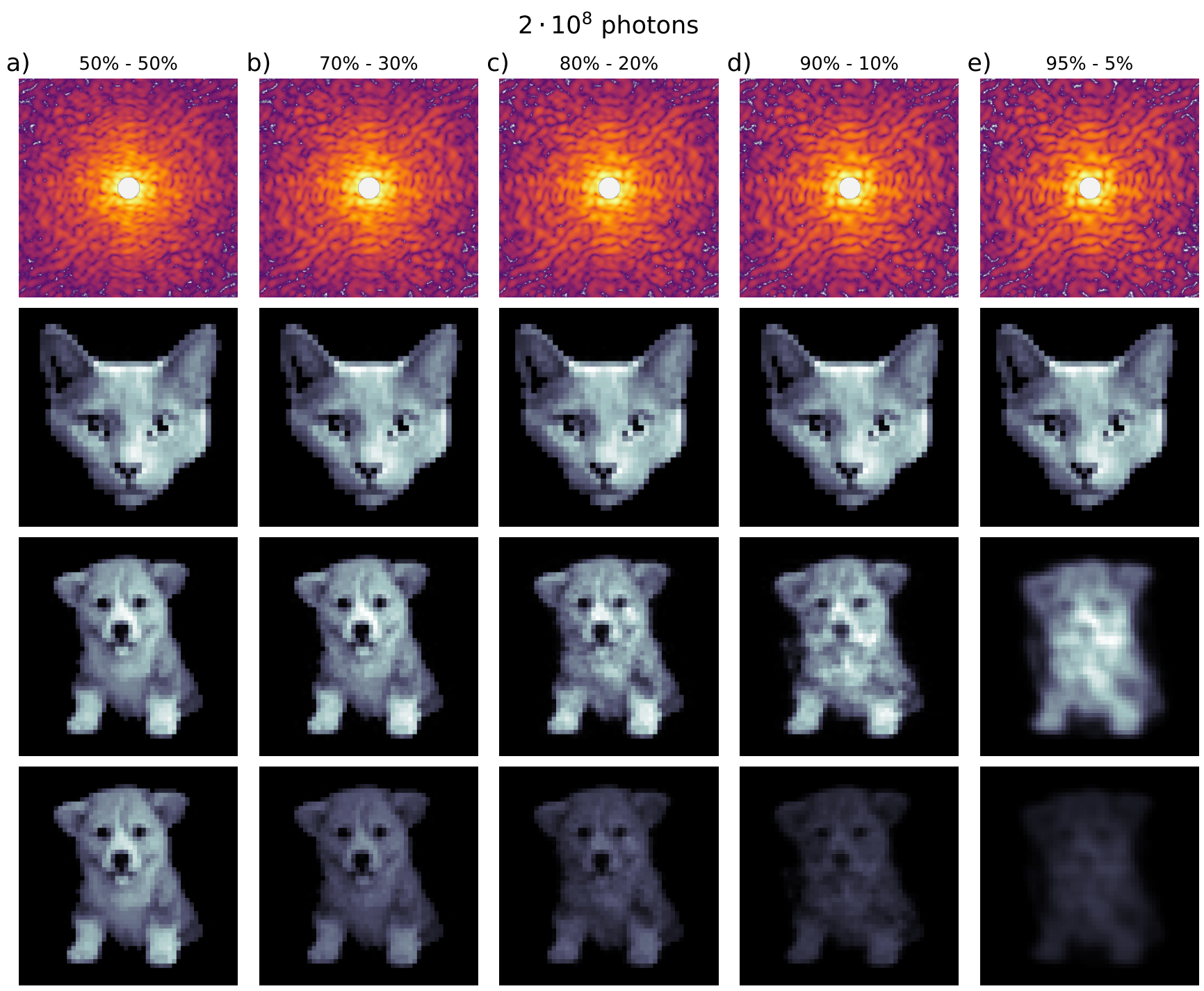

Figure S5: Impact of different relative brightness on the reconstruction for a total photon count of $2 \cdot 10^8$. The figure is organized as Fig. S4.

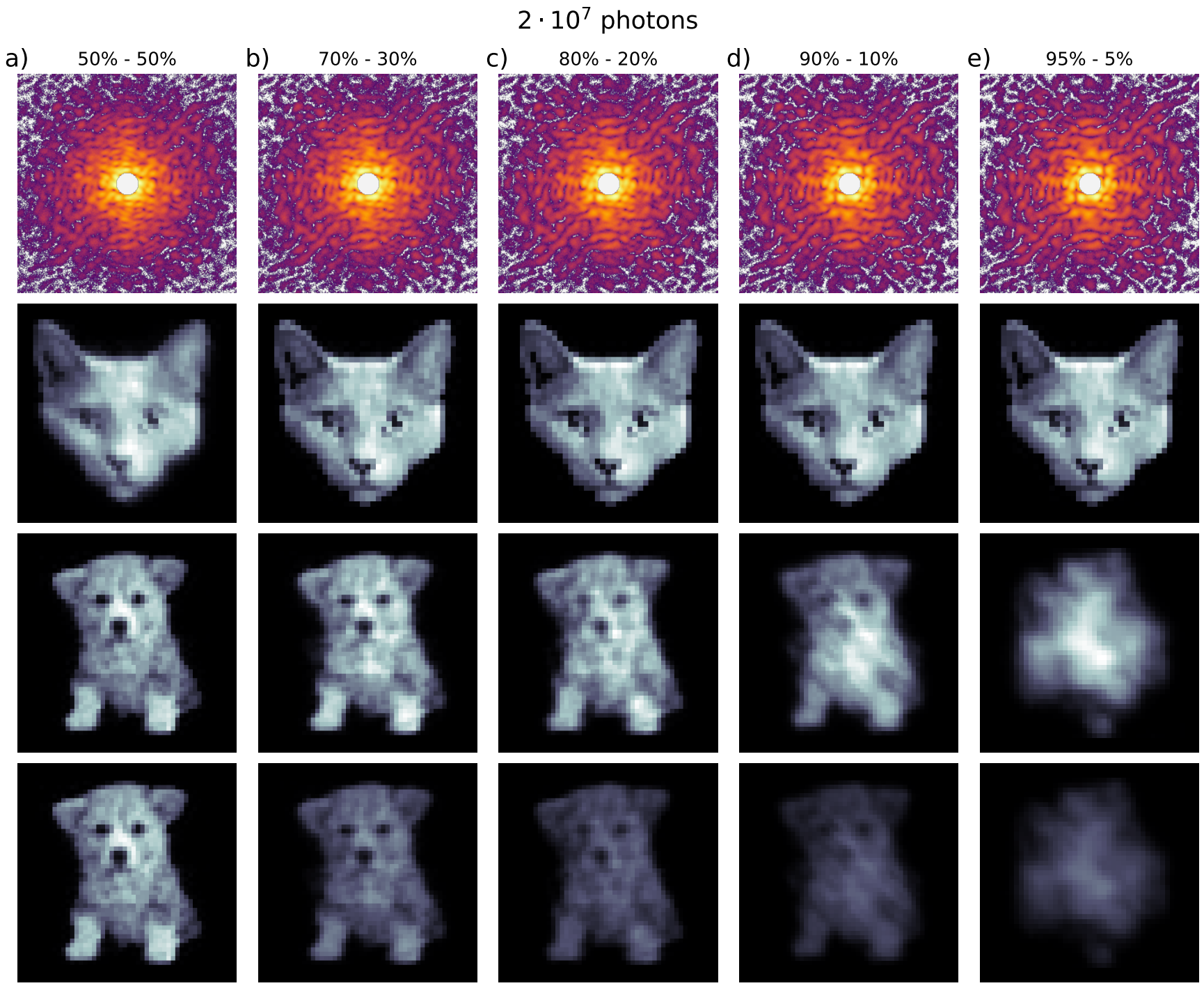

Figure S6: Impact of different relative brightness on the reconstruction for a total photon count of $2 \cdot 10^7$. The figure is organized as Fig. S4.

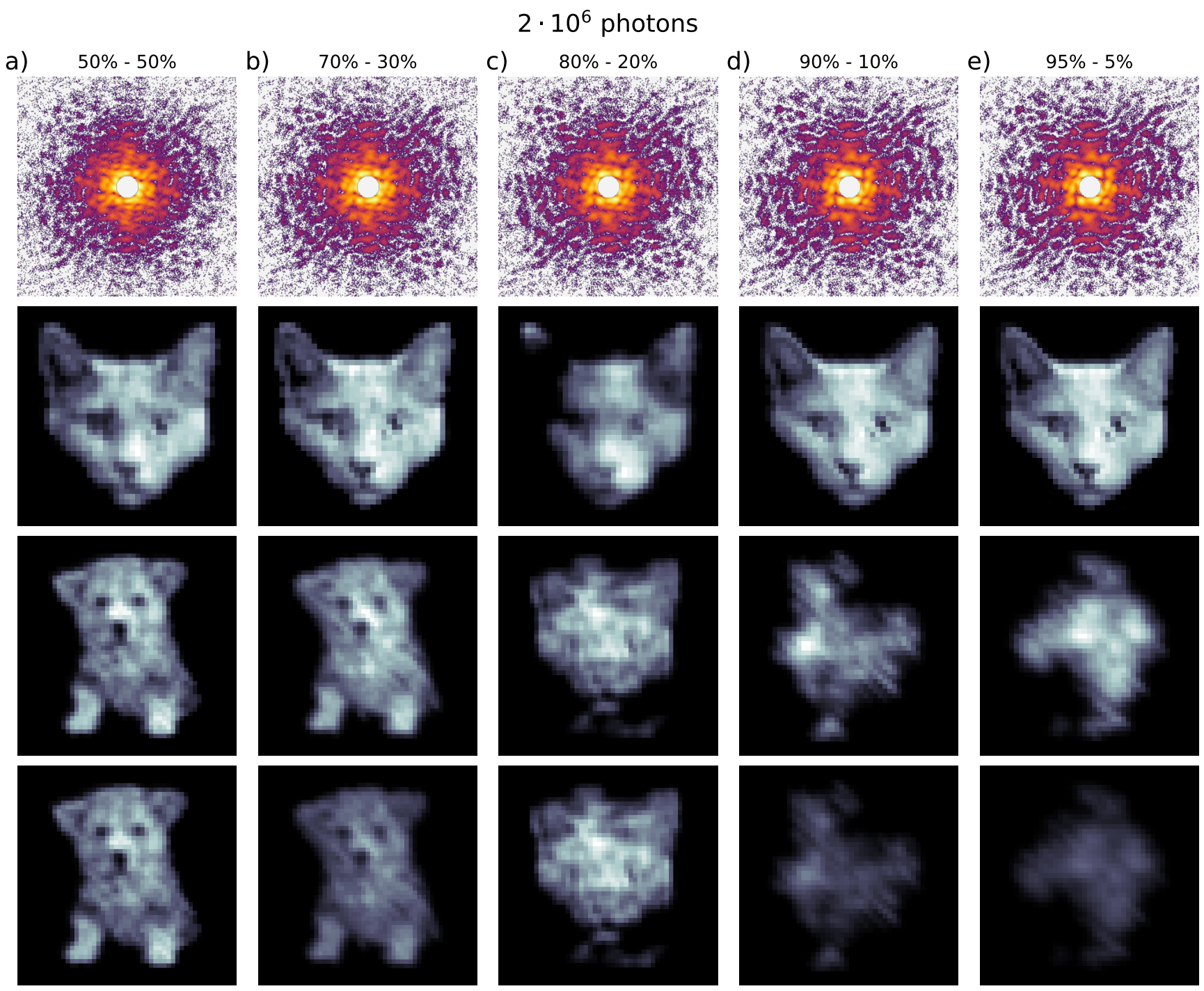

Figure S7: Impact of different relative brightness on the reconstruction for a total photon count of $2 \cdot 10^6$. The figure is organized as Fig. S4.

S1.4 Intensity dependence for Droplet-CDI

As discussed in Sec. III A of the main manuscript, the two-color operational mode of the XFEL comes with a reduction in the overall intensity of the two pulses, compared to the single-color mode. This feature is significantly impacting imaging experiments, which are inherently *photon-hungry*. Two-color patterns from xenon-doped superfluid helium nanodroplets allow dichrographic imaging only under optimal conditions for both FEL pulses using the Droplet-CDI approach [3]. In this section, we perform a systematic analysis on the performance of *Dichography* on simulated data, by tuning the overall brightness to better understand and evaluate the results presented in Sec. III A.

Simulations are performed for a superfluid helium nanodroplet with a diameter of $420\,\mathrm{nm}$. Inside the droplet, a structure resembling a filament of xenon aligned along a quantum vortex [4, 5] is added. The filament thickness is set to $5\,\mathrm{nm}$. The density of the materials is tuned to $0.125\,\mathrm{g/cm^3}$ for superfluid helium and $3.000\,\mathrm{g/cm^3}$ for xenon. The scattering cross sections of the two components have been adjusted accordingly.

Like the tests presented in Sec. S1.2, the performance of *Dichography* is probed for different numbers of total scattered photons $N_{\text{ph}}^{\text{tot}}$. The relative brightness between the two frames is adjusted to match the one measured for the experimental pattern presented in Fig. 2a of the main manuscript, where the signal from the $1.2\,\mathrm{keV}$ *probe* pulse scatters $64\,\%$ more photons than the $1.0\,\mathrm{keV}$ *pump* pulse. This is achieved by setting the photon balance parameter α to 0.38 (see Eq. (S2)). The structure of the xenon filament has been slightly modified between the two frames, to highlight wether the information is correctly separated.

The simulated patterns and the corresponding *Dichography* reconstructions are shown in Fig. S8a-g, for different total intensities. The intensity, indicated in terms of total scattered photons, can be quantitatively compared with the experimental data. In fact, from the experimental reconstructions in Fig. 2, the corresponding total yield of diffracted photons can be estimated to be $N_{\text{ph}}^{\text{tot}} = 2.3 \cdot 10^6$ for Fig. 2a and $N_{\text{ph}}^{\text{tot}} = 1.5 \cdot 10^6$ for Fig. 2d.

The first row (Fig. S8a) corresponds to a total photon yield, indicated at the right end, about 25 times higher than that observed in the experiment. The higher intensity can also be qualitatively deduced by visual comparison of the scattering image with those reported in Fig. 2a and 2d. At such a high photon count, the corresponding two reconstructed frames show very high quality, with sharp density profiles that also clearly highlight the high achieved resolution. The structural difference between the two frames is well identifiable, with the right end of the filament being more stretched and straighter in the $1.2\,\mathrm{keV}$ frame.

With the reduction in intensity down to $5 \cdot 10^6$ photons, in Fig. S8b and S8c, the structural properties of the two frames remain consistent. The lower number of photons affects mostly the outer area of the detector, reducing the actual resolution of the reconstructions. Due to this loss in resolution, the reconstructed xenon structure appears thicker with decreasing brightness.

The range of photon counts between $3 \cdot 10^6$ and $1.5 \cdot 10^6$, in Fig. S8d–f, corresponds to a transition region between reliable reconstruction conditions and situations that lead to reconstruction failure. This is also the range in which the experimental reconstructions shown in Fig. 2 lie, with $N_{\text{ph}}^{\text{tot}} = 2.3 \cdot 10^6$ and $N_{\text{ph}}^{\text{tot}} = 1.5 \cdot 10^6$, respectively.

These simulations give insight into how a reconstruction fails with reduced brightness. The first signs are fluctuations in the density signal along the filaments, with regions of higher or lower scattering cross section (Fig. S8d). Here, the filaments still retain their correct structure. At even lower photon yields (Fig. S8e), structural artifacts start to appear on top of the density fluctuations: the overall shape of the xenon agglomerate is still mostly present, but large density blobs arise along the filament line. The lowest brightness that still carries some structural information is reported in

Fig. S8f. There, the fluctuations in the density values of xenon are so strong that some parts of the structure are missing. The correspondence with the original structure is barely perceivable, but still present.

Finally, at a photon count of $1.0 \cdot 10^6$, most of the structural information is completely lost, with little or no direct connection between the original filament shapes and the retrieved densities, which show noisy artifacts distributed all around the droplet, with no structural correspondence even between the two frames.

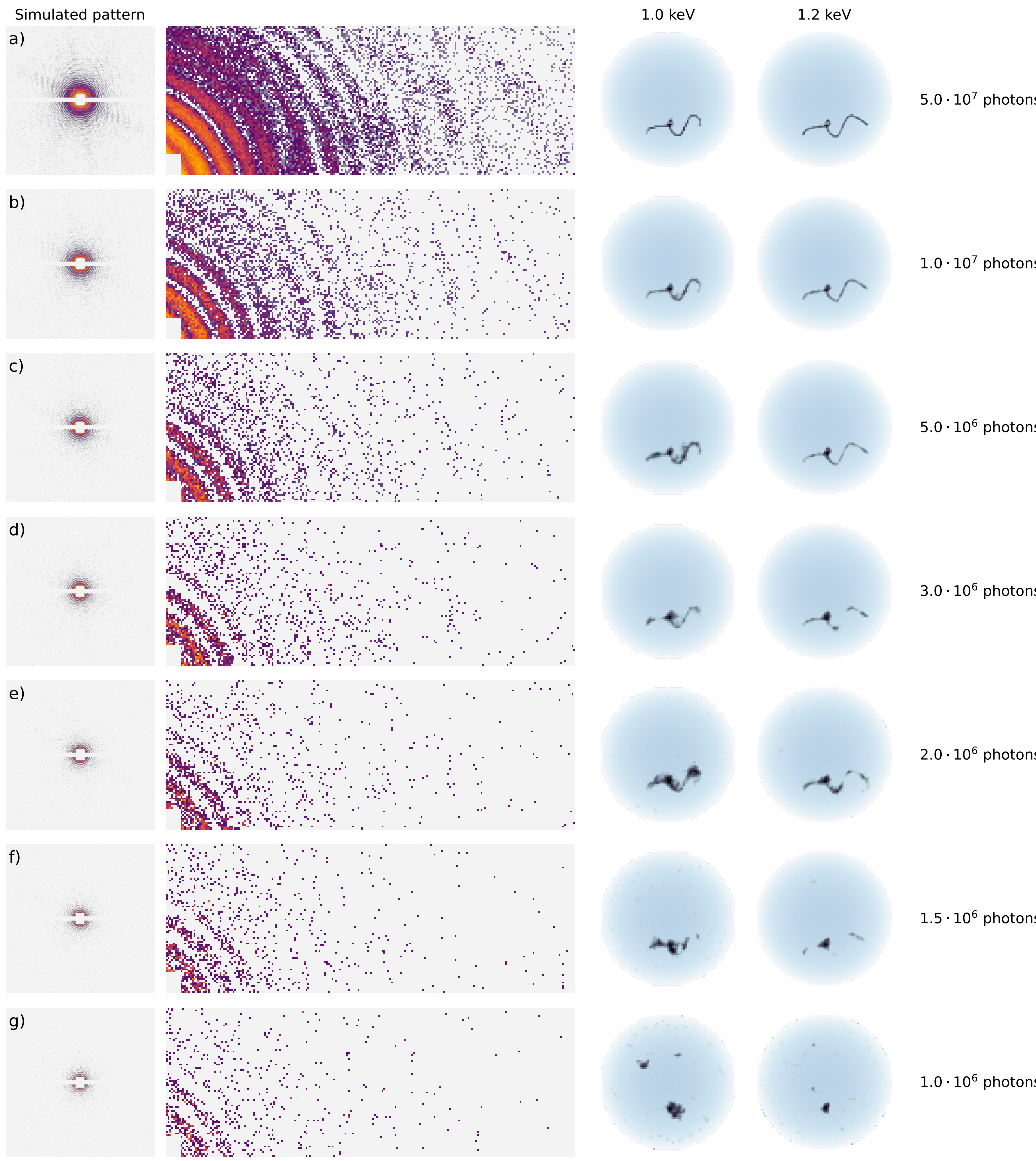

Figure S8: Impact of different brightness on the *Dichography* performance when combined with the DCDI method. Each row, from a) to g), deals with a scattering simulation of the same system, for different intensities of the recorded scattering signals. The first column corresponds to the simulated diffraction data, with a zoomed-in region reported in the second column, to better visualize the photon statistics. The third and fourth columns report the two frames of the *Dichography* reconstruction, for the $1.0\,\mathrm{keV}$ and the $1.2\,\mathrm{keV}$, respectively. At the end of each row, the total number of scattered photons is indicated.

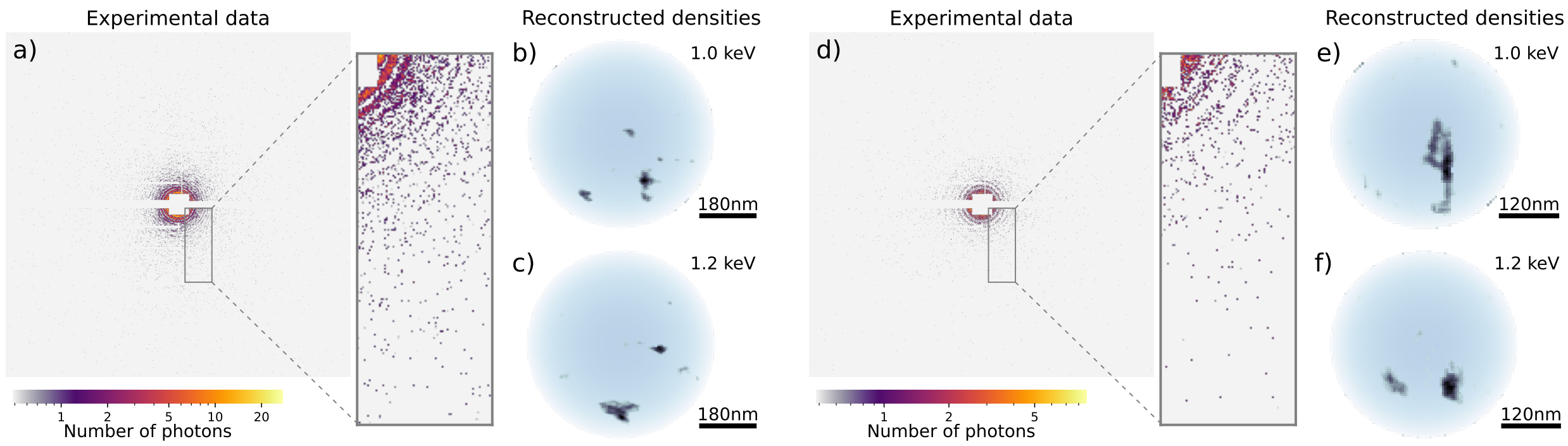

Figure S9: Examples of failed reconstructions from two-color diffraction patterns. The experimental diffraction patterns are reported in a) and d), on which the diffraction signals produced by two different photon energies, $1.0\,\mathrm{keV}$ and $1.2\,\mathrm{keV}$, overlap. The intensity is encoded as reported by the color bar at the bottom. The intensity values are translated into numbers of $1.2\,\mathrm{keV}$ photons per pixel. The inset plot is a zoomed-in region of the diffraction image, to better appreciate the photon statistics, and thus the statistical noise, that affects the experimental data. The two reconstructed frames of the electronic density are reported in b), c) and e), f) for the patterns in a) and d), respectively. In particular, b) and e) report the reconstruction from the $1.0\,\mathrm{keV}$ pulse, while c) and f) correspond to the $1.2\,\mathrm{keV}$ pulse.

S1.5 Examples of failed two-color reconstructions

In this section we show additional reconstructions of two-color data, where imaging failed, i.e., where the structures reconstructed in the two frames, corresponding to the two views on the sample by different colors, are inconsistent. Examples of failed reconstructions are shown in Fig. S9. It is worth noting that the features of the reconstructed frames, in Fig. S9b and Fig. S9c for the pattern in Fig. S9a, and in Fig. S9e and Fig. S9f for the pattern in Fig. S9d, closely resemble what observed in the simulated data for the low photon yield in Fig. S8g. Fig. S9 aims at giving a more consistent view on the reconstruction quality, and at making the reader more aware of the significance of the results presented in Fig. 2 of the main manuscript, despite their low signal-to-noise ratio and the presence of artifacts.

S2 Statistics and data selection of the two-color experiment

The two-color experiment on xenon-doped helium nanodroplets presents additional challenges regarding data selection. In a typical CDI experiment, diffraction data suitable for imaging can be selected in a relatively straightforward manner by evaluating the overall brightness of the recorded patterns. However, this selection step alone is insufficient for two-color experiments, as a further necessary requirement is that the two colors contribute comparably to the scattering signal.

For a single pattern, the intensity ratio between the two colors varies significantly shot-by-shot due to the different positions of the two foci along the beam propagation direction [6]. Consequently, the actual field intensities depend strongly on the position of the droplet in the interaction region. In particular, the *effective hit rate* of a two-color experiment is significantly lower than the overall hit rate, because only a fraction of the total focal volume provides similar intensities for both colors.

As discussed in the main text, the two-color reconstruction is based on an adaptation of the DCDI method [3] to Dichography. The DCDI approach requires extracting the helium droplet size and the corresponding scattering intensities of the two colors by fitting the experimental data. The fitting procedure involves four parameters:

- D/λ_{1keV} and $D/\lambda_{\text{1.2keV}}$: the droplet size normalized by the wavelengths of the two colors;
- N_{1keV} and $N_{\text{1.2keV}}$: the normalization factors of the two scattering contributions of the droplet.

An effective method to obtain an initial selection of patterns containing significant signal from both colors is to perform the fitting without constraining the ratio of the two wavelengths. A retrieved ratio between the two photon energies close to the nominal value of $\frac{1.2\,\text{keV}}{1\,\text{keV}} = 1.2$ acts as a clear indicator that scattering from both colors is present in the pattern in a significant amount, sufficient to carry structural information. This initial analysis, performed on the 673 available patterns with usable signal, is reported in Fig. S10a.

A second selection step is then performed on those patterns with a detected photon energy ratio between 1.15 and 1.25 with sufficient brightness, i.e., within the blue square in Fig. S10a. This second analysis is reported in Fig. S10b, and it takes into account two aspects discussed in the following.

On the one hand, the "information content" of the scattering has to be the highest possible. We can then define a normalized brightness of the pattern I^N as:

$$I^N = \frac{\sum I_{ij}}{D^2} \tag{S7}$$

where D is the retrieved particle size. Under a numerical point of view, it defines the information recorded per pixel of the sample to be reconstructed. Indeed, the information to be retrieved via imaging scales with the *support* size, related to the geometrical cross section of the sample, which scales proportionally to D^2. Larger samples require a brighter pattern to reach the same reconstruction quality. The quantity I^N is shown on the y-axis of Fig. S10b.

On the other hand, it is necessary that the "brightness" of the real-space representation of the droplet density is similar between the two colors. This information can be calculated from the density normalizations $N_{\text{1.2keV}}$ and $N_{\text{1.keV}}$ provided by the fitting. In particular, the number of scattered photons per real-space pixel can be calculated. The ratio of the number of scattered photons per pixel between the two colors is shown on the x-axis in Fig. S10b.

The optimal patterns in Fig. S10b are those that are both close to an equal contribution to the scattering signal (vertical dashed line) and are in the upper part of the plot. These are, indeed, the

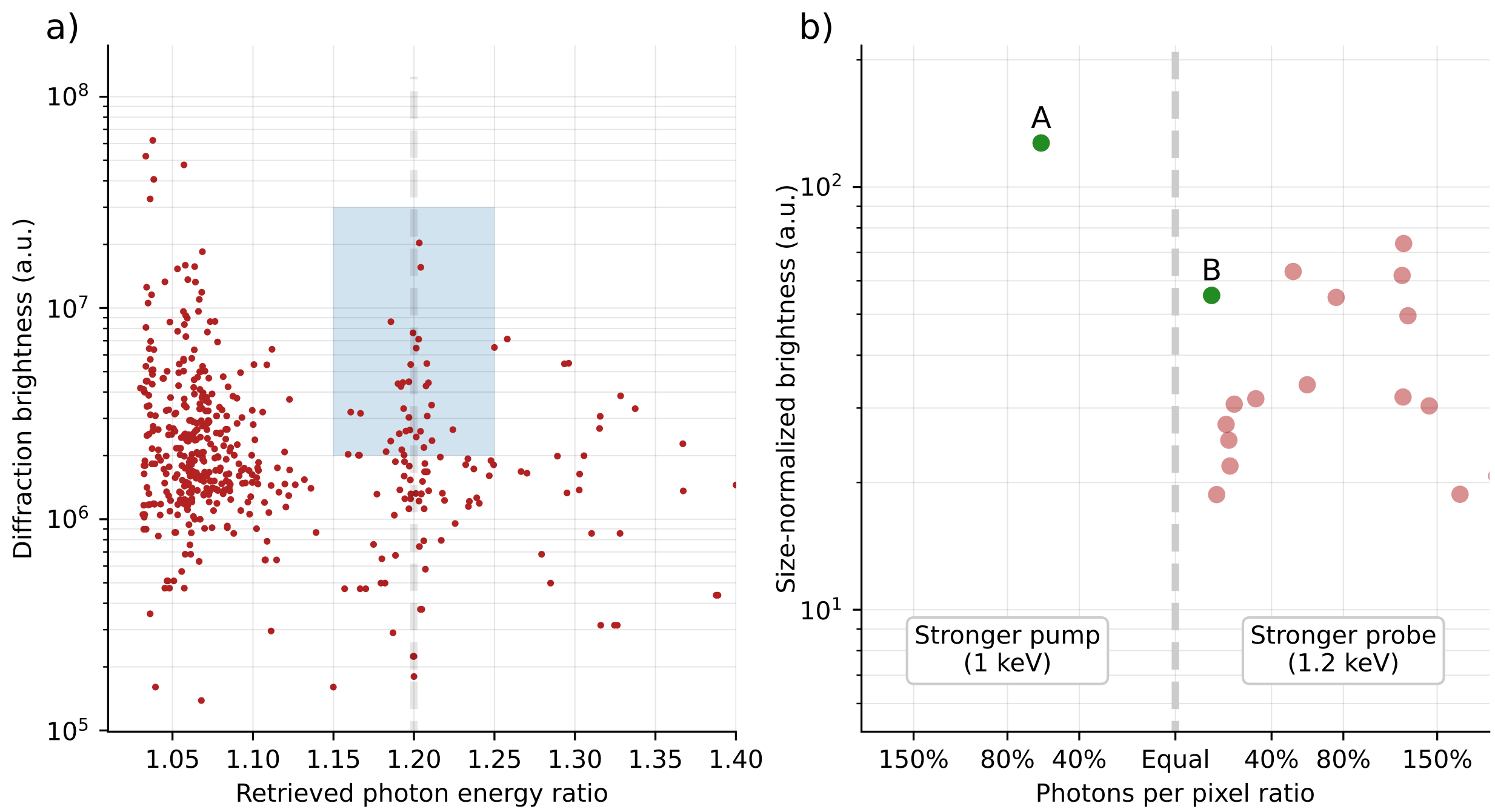

Figure S10: Selection of two-color diffraction patterns for imaging. In a), each red dot corresponds to an individual diffraction image, with the overall brightness of the pattern indicated on the y axis. The x axis shows, instead, the ratio between photon energies retrieved by fitting. The blue rectangle shows the region where patterns have both enough brightness and a retrieved photon energy ratio close to the nominally expected value of 1.2, indicated with the grey dashed line. The patterns within the blue rectangle are then selected for further analysis, reported in b). Here, the pattern brightness normalized by the geometrical cross section (y-axis) of the sample is shown against the ratio of the scattering contribution of the two colors, as retrieved by the fitting. The grey dashed line indicates the optimal condition, i.e., where the two colors equally contribute to the overall scattering signal. The two dots in green, marked as A and B, correspond to the two patterns shown in Fig. 2a and Fig. 2b of the main manuscript, respectively.

properties of the two reconstructions shown in Fig. 2 of the main paper, highlighted in Fig. S10b as green dots.

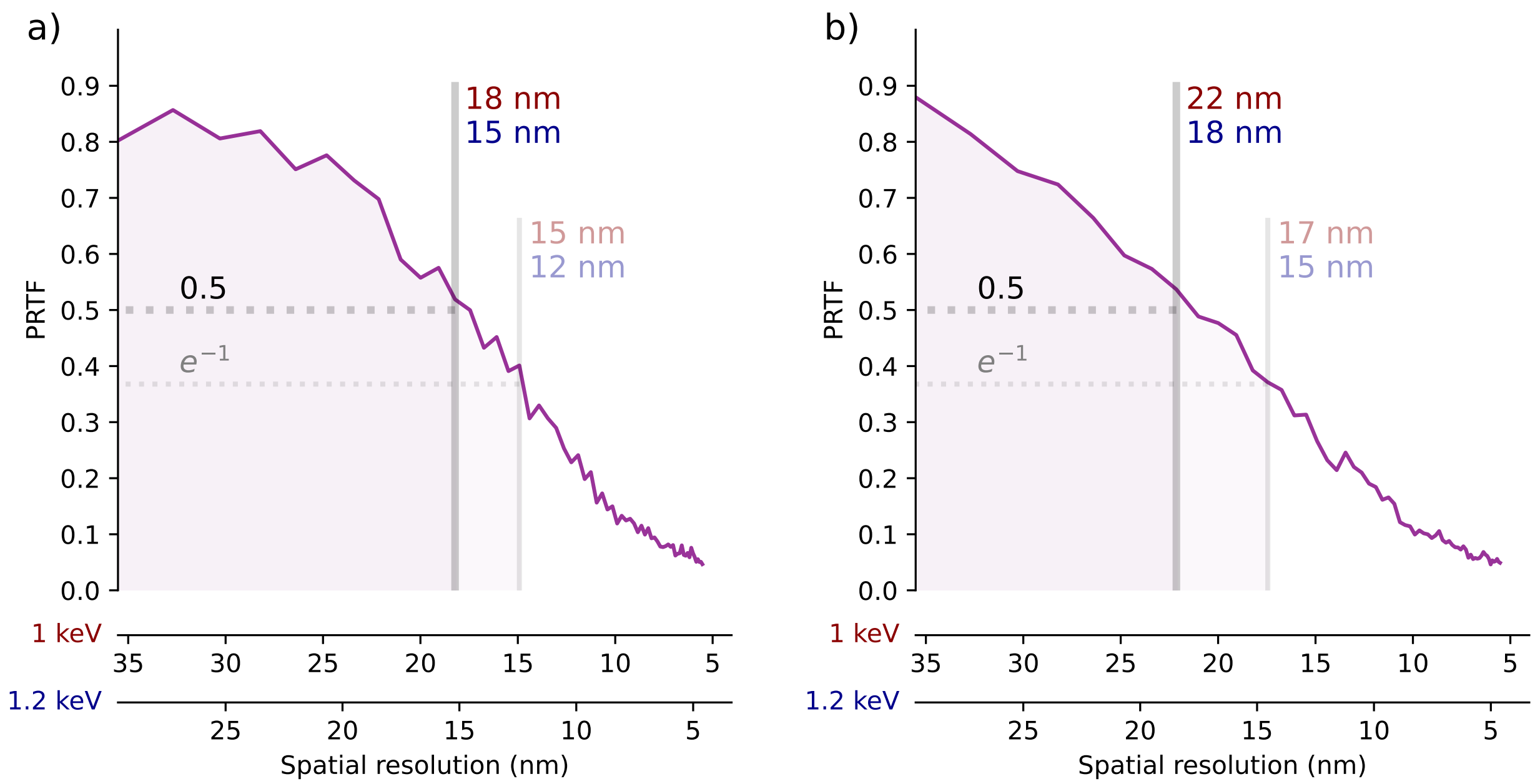

Figure S11: Spatial resolution achieved by the two-color reconstructions shown in Fig. 2 of the main paper, calculated via the Phase Retrieval Transfer Function (PRTF). Half-period resolutions are shown both for a PRTF value of 0.5 and $1/e$. Due to the low signal to noise ratio in the diffraction data we prefer to be conservative and consider as resolution the value provided by PRTF $= 0.5$. As the conversion of the recorded momentum transfer to spatial resolution depends on the photon energy, we indicate the corresponding resolution in red color for the $1\,\mathrm{keV}$ pulse (pump), and in blue color for the $1.2\,\mathrm{keV}$ pulse (probe).

S2.1 Spatial resolution of two-color reconstructions

We estimate the spatial resolution of the two-color results in Fig. 2 of the main paper via the Phase Retrieval Transfer Function (PRTF) [7]. The PRTF analysis is reported in Fig. S11.

While the PRTF is an established and robust method to evaluate the resolution of conventional CDI reconstructions, its meaning is not straightforward when applied to *Dichography*. Indeed, the PRTF cannot disentangle the two contributions from the two frames, and consequently it is not possible to directly assign an individual resolution to the two reconstructed images. Furthermore, the resolution cannot be uniquely defined. Indeed, due to the different photon energies, the recorded signal at a specific scattering angle corresponds to two different momentum transfers, and thus to two different spatial resolutions depending on the X-ray color.

S3 Spatial separation for double-hit patterns

When two particles are simultaneously present in the interaction region, they both scatter their own field. The two fields interact, introducing an interference term in the recorded data. However, the frequency of the interference term is proportional to the spatial separation between the particles: the larger the distance, the higher the oscillation frequency of the interference. When the signal oscillation of the interference is smaller than the sampling capabilities of the detector, this part of the signal is lost. In this case, the data can be employed for *Dichography*, as discussed in Appendix III B of the main manuscript in the case of silver nanoparticles investigated at SwissFEL.

Here, we simulate the diffraction signal for different distances between the nanoparticles in the direction transverse to the FEL propagation axis, and we show under which conditions the interference term is captured, or not, by the detector. A first example is given in Fig. S12, for a 2048$\times$2048 pixel detector. This simulation is similar to the actual experimental conditions, where the 4 megapixel Jungfrau detector is employed [8]. To match the experiment, also the photon energy is adjusted to $1\,\mathrm{keV}$ and the maximum acquired scattering angle is set to $13.5°$. Fig. S12 shows, in addition to the diffraction pattern for the different distances and a zoomed region to better highlight the interference signal, also the autocorrelation function of the sample. This can be obtained via a direct Fourier Transform (FT) of the simulated scattering.

The autocorrelation function is a precious indicator, as it makes straightforward to recognize if the interference term is contained, or not, in the recorded data. In particular, the central peak of the autocorrelation is the convolution of the individual particles with themselves. The two additional peaks come, instead, from the cross-correlation between one particle and the other, and it can only appear if the interference between the two particles is correctly sampled. Fig. S12 shows that, employing the full-resolution data, interference is resolved up to $3.5\,\mu\mathrm{m}$.

Fig. S13 and Fig. S14 are the same simulations performed instead for detectors with 1024$\times$1024 and 512$\times$512 pixel dimensions, with the other experimental parameters unchanged. A reduction of the sampling capabilities impacts the maximum distance at which interference is resolved. For example, for a 1024$\times$1024 pixel detector, interference is already lost at a distance of $2\,\mu\mathrm{m}$. This distance reduces to less than $1\,\mu\mathrm{m}$ for a 512$\times$512 pixel count.

All double-hit reconstructions of silver nanoparticles used a diffraction pattern rescaled to 512$\times$512 via a binning operation, artificially reducing the sampling capability of the detector. So, in this case, a separation of $1\,\mu\mathrm{m}$ between the particles suffices to avoid the interference term and to make the data compatible with *Dichography*.

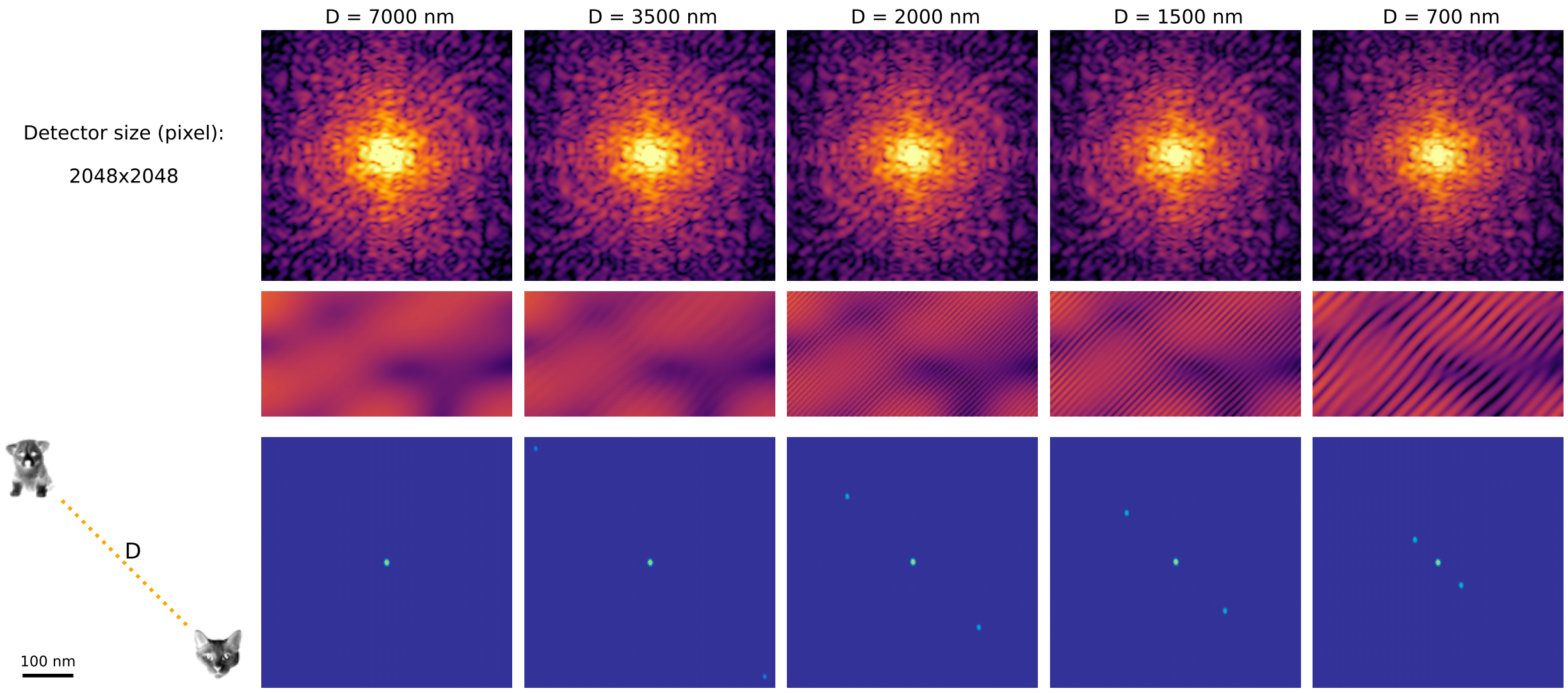

Figure S12: Simulation of a double-hit pattern as a function of the particle distance D for a 2048$\times$2048-pixel detector, with geometry and photon energy equivalent to those of the Swiss-FEL experiment. Each column corresponds to a different distance between the two particles. The first row shows the simulated diffraction pattern. The second row displays a cropped region of the pattern to make the interference signal more visible. The last row shows the autocorrelation function of the system, obtained by an inverse Fourier transform of the pattern.

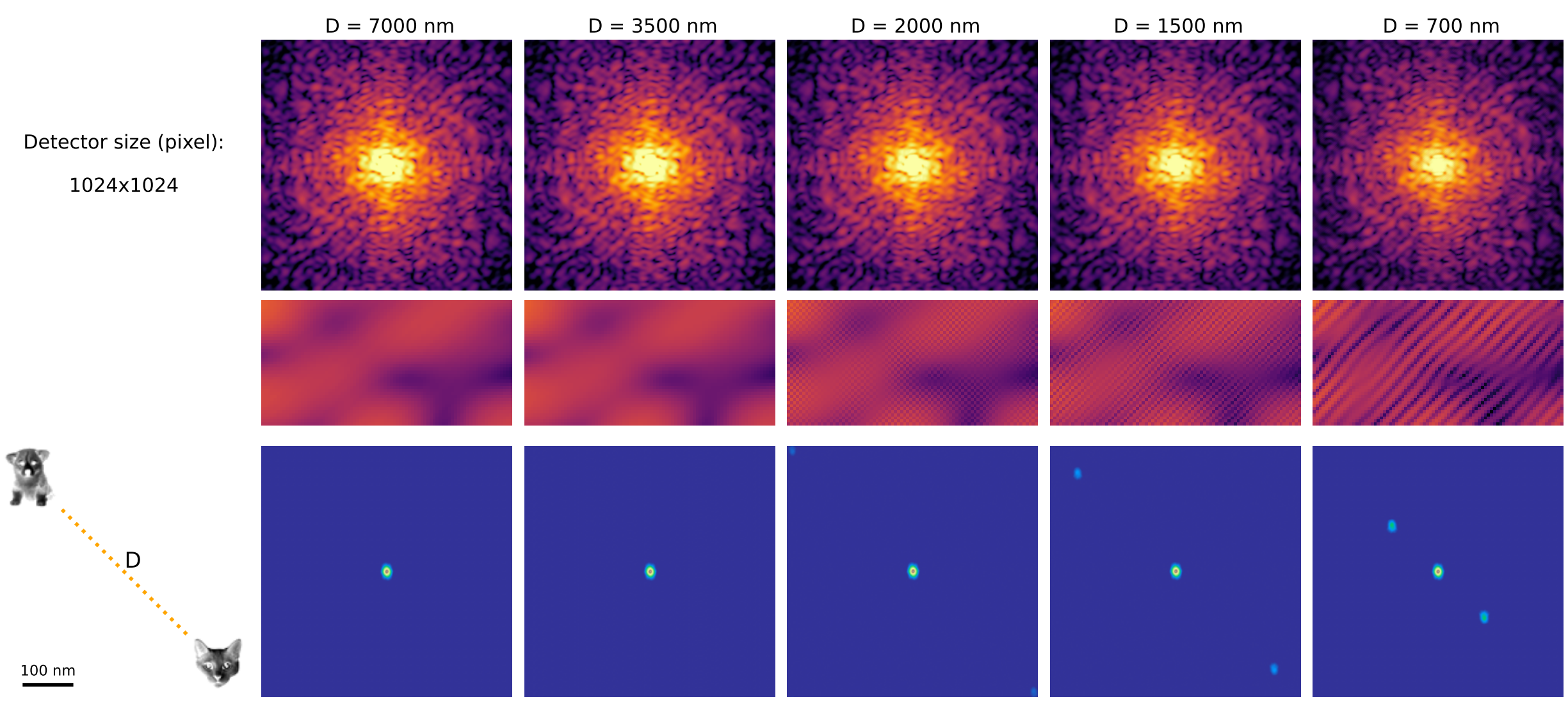

Figure S13: Simulation of a double-hit pattern as a function of the particle distance D for a 1024$\times$1024-pixel detector, with geometry and photon energy equivalent to those of the Swiss-FEL experiment. Each column corresponds to a different distance between the two particles. The first row shows the simulated diffraction pattern. The second row displays a cropped region of the pattern to make the interference signal more visible. The last row shows the autocorrelation function of the system, obtained by an inverse Fourier transform of the pattern.

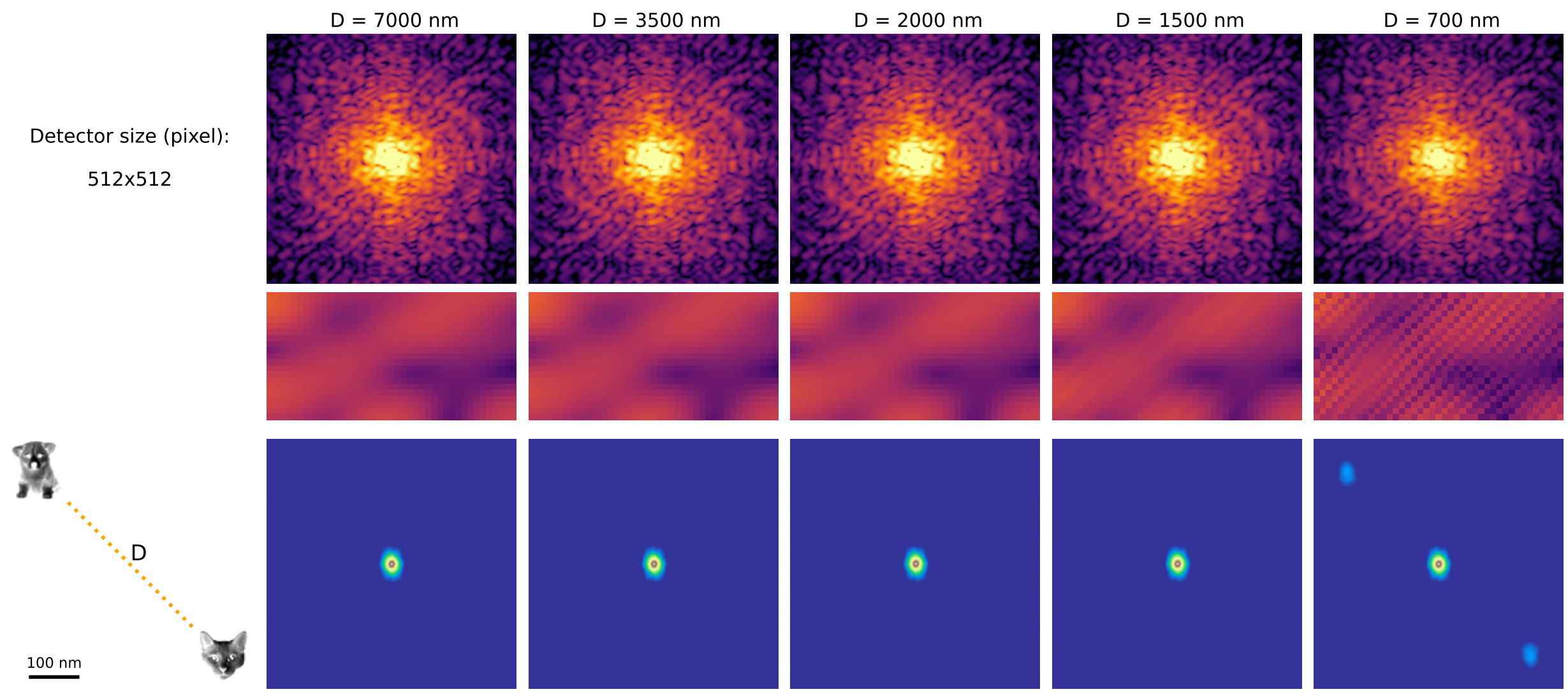

Figure S14: Simulation of a double-hit pattern as a function of the particle distance D for a 512$\times$512-pixel detector, with geometry and photon energy equivalent to those of the SwissFEL experiment. Each column corresponds to a different distance between the two particles. The first row shows the simulated diffraction pattern. The second row displays a cropped region of the pattern to make the interference signal more visible. The last row shows the autocorrelation function of the system, obtained by an inverse Fourier transform of the pattern.

S4 The intensity constraint

Dichography employs an adapted version of the *intensity projector* of conventional CDI [9], as introduced in Appendix VI A 2 of the main text. Here we present a more formal definition of the mathematical operations that are used to *back-propagate* the experimental information to the two frames of the reconstruction [10].

Given ψ_{ij}^{A} and ψ_{ij}^{B} the matrices containing the current field estimates of ρ^{A} and ρ^{B}, obtained with a Fourier Transform (FT) operation, we define the tensor $\mathcal{V} \in \mathbb{C}^2$. Its entries at coordinates i, j are defined as:

$$\mathcal{V}_{ij} = \begin{pmatrix} \psi_{ij}^{A} \\ \psi_{ij}^{B} \end{pmatrix} \tag{S8}$$

In simple terms, the tensor $\mathcal{V}$ is formed by stacking the two fields ψ_{ij}^{A} and ψ_{ij}^{B}. Eq. (S8) allows us to express the condition in Eq. (2) of the main text in the following, formal but compact, form:

$$\|\mathcal{V}_{ij}\| = \sqrt{I_{ij}} \tag{S9}$$

where $\|\cdot\|$ indicates the L^2 norm.

The enforcing of the Fourier intensity constraint can be thus reduced to a trivial renormalization of the entries in the matrix $\mathcal{V}_{ij}$. Given $\mathcal{V}$ containing the current field estimates ψ^{A} and ψ^{B}, a new tensor $\mathcal{V}'$ can be created as:

$$\begin{aligned} \mathcal{V}'_{ij} &= \frac{\mathcal{V}_{ij}}{\|\mathcal{V}_{ij}\|} \cdot \sqrt{I_{ij}} \\ &= \begin{pmatrix} \frac{\psi_{ij}^{A}}{\sqrt{\left|\psi_{ij}^{A}\right|^2 + \left|\psi_{ij}^{B}\right|^2}} \cdot \sqrt{I_{ij}} \\ \frac{\psi_{ij}^{B}}{\sqrt{\left|\psi_{ij}^{A}\right|^2 + \left|\psi_{ij}^{B}\right|^2}} \cdot \sqrt{I_{ij}} \end{pmatrix} \\ &= \begin{pmatrix} \psi_{ij}^{A\prime} \\ \psi_{ij}^{B\prime} \end{pmatrix}. \end{aligned} \tag{S10}$$

It is straightforward to verify that, for each i, j, $\mathcal{V}'_{ij}$ satisfies the condition in Eq. (S9), i.e., its components $\psi_{ij}^{A\prime}$ and $\psi_{ij}^{B\prime}$ fulfill Eq. (2). In simple terms, the magnitude of each of the two fields ψ_{ij} is corrected by a factor $\frac{\sqrt{I_{ij}}}{\sqrt{\left|\psi_{ij}^{A}\right|^2 + \left|\psi_{ij}^{B}\right|^2}}$. It is, furthermore, trivial to verify that the operation in Eq. (S10) is a *projection* operation, thus retaining the mathematical properties of its counterpart in conventional CDI [9].

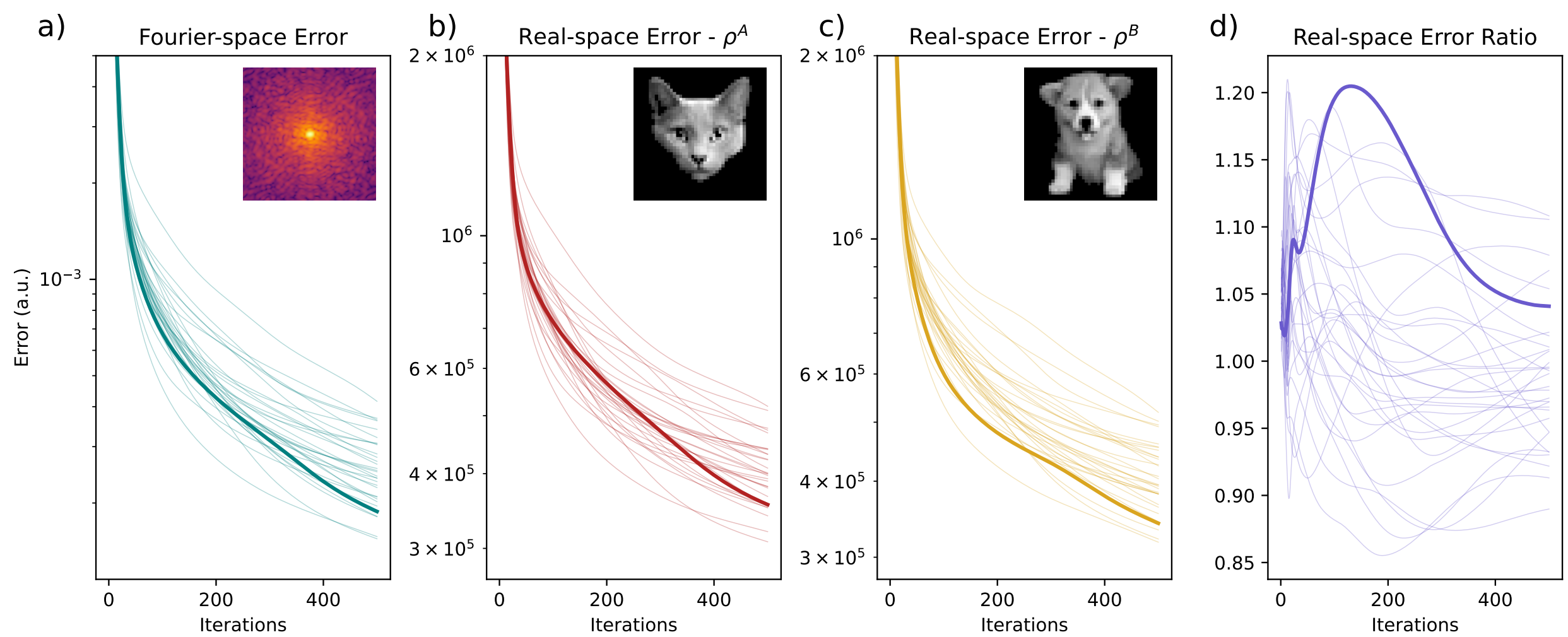

Figure S15: Typical behavior of the error value for the *dichographic* version of the Error Reduction algorithm is reported for 32 different reconstruction attempts of 500 iterations each. The error value computed in Fourier space is plotted in a). Instead, b) and c) report the error value calculated in real space, which is separated for the two frames. The ratio between the real-space errors of ρ^A and ρ^B is plotted in d), to underline how the two frames can differently contribute to the overall error value. The thick line in each plot highlights the behavior of a single, randomly selected, reconstruction process.

S5 Behavior of the iterative algorithms

The modified *intensity constraint* described in Appendix VI A 2 of the main text allows for a straightforward adaptation of the conventional iterative phase retrieval (IPR) algorithms [9] to *Dichography*. As reported in Appendix VI A 1, the reconstruction of the two frames is built upon two separate IPR procedures, which are entangled via the intensity constraint in Fourier space while remaining independent in real space through the application of the *support constraint*.

Among the different IPR algorithms that can be implemented, the Error Reduction algorithm (ER) is of particular importance for conventional CDI. It takes its name from the fact that it is a *steepest-descent* method for the error function, i.e., the error value is always reduced at each iteration [11]. The *Dichography* intensity constraint retains the original property of being a *projection* operation [9]. However, the link between the real space, where the support function is constrained, and the reciprocal space, where the experimental intensities are constrained, is no longer a plain Fourier transform. One implication is that the norm is not preserved and, thus, the ER algorithm loses its strictly *steepest-descent* characteristics in the *dichographic* version.

In this section, we investigate the behavior of the error with proceeding algorithm iterations and highlight some peculiarities of the iterative phase retrieval algorithms adapted to *Dichography*.

The error value is evaluated as the difference between the recorded diffraction pattern and the current estimate of the field. For *Dichography*, the error of a reconstruction for two given frames ρ^A and ρ^B can be evaluated as:

$$\mathcal{E}[\rho^A, \rho^B] = \left\| \sqrt{|\mathcal{F}[\rho^A \cdot S^A]|^2 + |\mathcal{F}[\rho^B \cdot S^B]|^2} - \sqrt{I} \right\| \tag{S11}$$

where S^A, S^B are the support functions of the two frames, and the operations $\rho \cdot S$ set to zero the

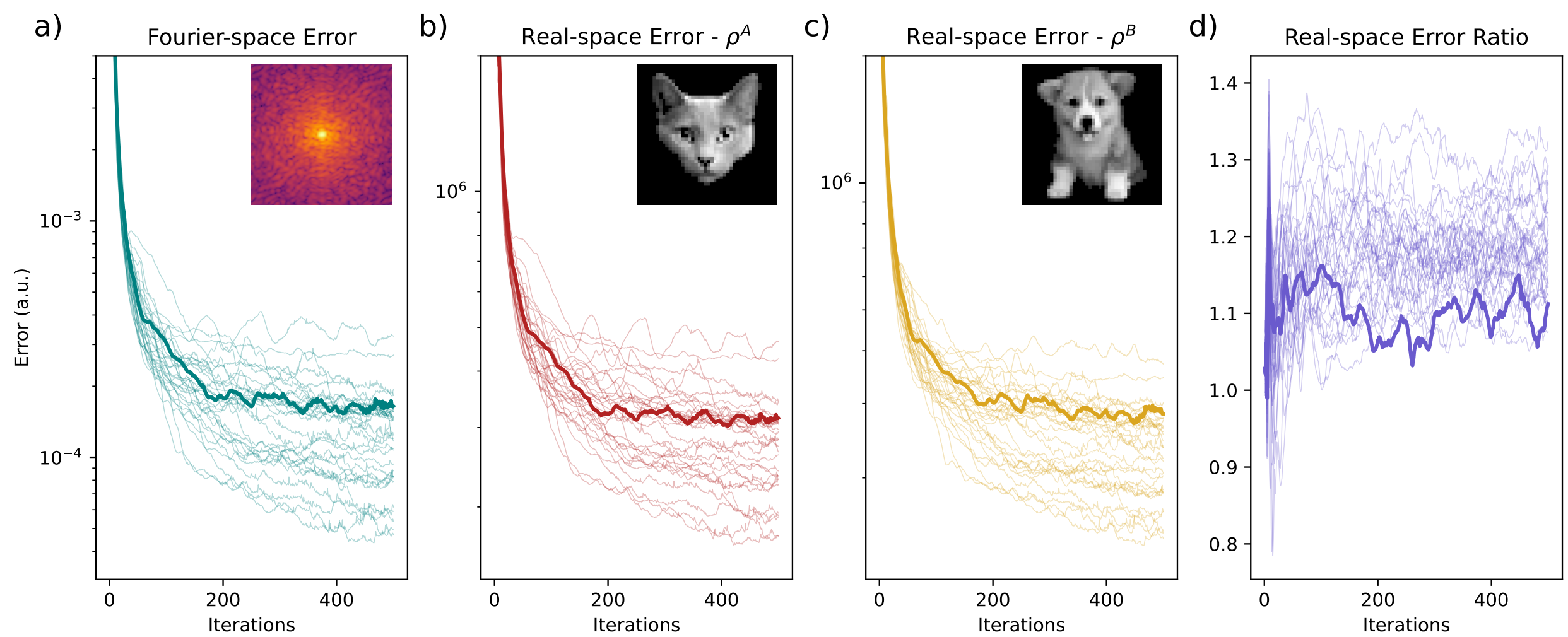

Figure S16: Typical behavior of the error value for the *dichographic* version of the Hybrid Input-Output algorithm, reported for 32 different reconstruction attempts of 500 iterations each. The error value computed in Fourier space is plotted in a). Instead, b) and c) report the error value calculated in real space, which is separated for the two frames. The ratio between the real-space errors of ρ^A and ρ^B is plotted in d), to underline how the two frames can differently contribute to the overall error value. The thick line in each plot highlights the behavior of a single, randomly selected, reconstruction process.

density values outside the support function. The operation in Eq. (S11) calculates in Fourier space the Euclidean distance (L^2 norm) between the current estimate and the experimentally measured scattering amplitude. The typical error behavior with ER iterations observed in *Dichography* is reported in Fig. S15a. An interesting feature of the reported error value is that it is systematically reduced over iterations, i.e., the algorithm behaves here as a *steepest-descent* method, despite this not being mathematically ensured.

A further error value can be calculated in real space by computing the L^2 norm of those coordinates outside the support constraint. For conventional CDI, the two ways of calculating the error are equivalent because of the *Parseval theorem*, as the FT operation preserves the L^2 norm. For *Dichography*, the link between the two spaces is more than a plain FT, and this strict link no longer holds.

In fact, in this case the error in real space can provide more insight, as the contributions to the error value from the two frames ρ^A and ρ^B can be independently computed. The behavior of the *real-space* error is reported in Fig. S15b and Fig. S15c for ρ^A and ρ^B, respectively. One of the 32 error traces, coming from 32 independent reconstructions, is highlighted to show the typical overall behavior. The real-space errors in Fig. S15b and Fig. S15c reveal that the error for both frames is decreasing, but their behaviors – and thus their contributions to the total error (Fig. S15a) – can differ and change during the process. This aspect is better illustrated in Fig. S15d, where the ratio between the two real-space errors of ρ^A and ρ^B is reported. Fluctuations of the ratio away from 1 indicate an imbalance in the error contributions. It is worth underlining that the error ratio in Fig. S15d, while related to the relative signal strength, cannot be trivially linked to the overall intensity ratio of the two frames; rather, it shows how the two frames contribute differently to the overall error as a function of the iterations.

A similar investigation of the error behavior can be accomplished in the case of the *dicho-*

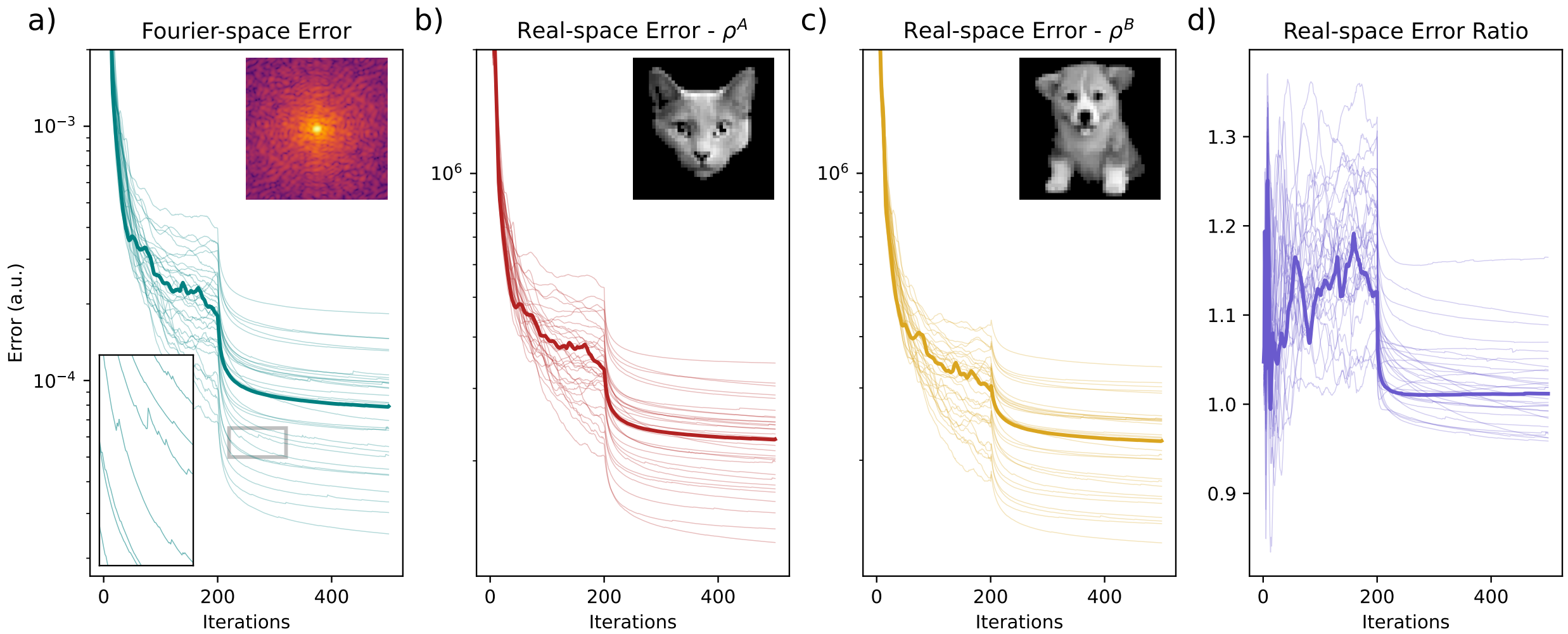

Figure S17: Typical behavior of the error value for a combination of 200 iterations of HIO followed by 300 iterations of ER algorithm, reported for 32 different reconstruction attempts. The error value computed in Fourier space is plotted in a), wherein the inset plot is a zoom-in of the region highlighted by the gray rectangle. Instead, b) and c) report the error value calculated in real space, which is separated for the two frames. The ratio between the real-space errors of ρ^A and ρ^B is plotted in d), to underline how the two frames can differently contribute to the overall error value. The thick line in each plot highlights the behavior of a single, randomly selected, reconstruction process.

graphic adaptation of the Hybrid Input-Output (HIO) algorithm [11], and is reported in Fig. S16. The HIO behavior retains the properties observed in conventional CDI, with a higher capability of reducing the error value when the reconstruction is far from the solution. In fact, the error reached by ER in Fig. S15 is significantly higher than the one reached by HIO after the same number of iterations.

The well-known drawback of the HIO algorithm is, however, its instability, visible in the strong fluctuations of the error value in Fig. S16. The usual approach in conventional CDI is to start a reconstruction attempt with the HIO algorithm, and then use the ER algorithm to improve the convergence properties. The same approach is undertaken in the error analysis shown in Fig. S17, where the first 200 iterations are performed with the HIO algorithm, and the following ones with the ER algorithm.

A first consideration regards the overall error value reached after 500 iterations, which is lower than both the ER-only attempt (Fig. S15) and the HIO-only attempt (Fig. S16), confirming that alternating the two algorithms is a more convenient approach, similar to what has been observed for conventional CDI.

A second note concerns the zoomed-in region reported in Fig. S17a, where a localized increase in the error value is observed despite the use of the ER algorithm. This behavior confirms that the ER algorithm loses its *steepest-descent* feature, as the error value can actually increase with subsequent iterations. However, we empirically observe that the increase in the error during ER iterations only occurs locally, and in practice the algorithm appears to retain its *steepest-descent* properties over the scale of several iterations.

S6 Conventional single-particle patterns with Dichography

This section reports the behavior of Dichography when reconstructing a conventional single-particle, single-color diffraction pattern. While the presence of scattering signals from both pulses is known *a priori* for the two-color experiment, this test further highlights the specific nature of the dichographic problem and clarifies its distinction from conventional Coherent Diffraction Imaging (CDI).

A single-particle diffraction pattern, which possesses a unique solution in the context of conventional CDI, yields infinite solutions when treated as a dichographic problem. Given an experimentally recorded single-particle intensity I^{sp} and the corresponding reconstructed density ρ^{sp}, the problem can be formulated as:

$$
\begin{aligned}
I^{\text{sp}} &= \alpha\, I^{\text{sp}} + (1-\alpha)\, I^{\text{sp}} \\
&= \alpha \left|\mathcal{F}\left[\rho^{\text{sp}}\right]\right|^2 + (1-\alpha)\left|\mathcal{F}\left[\rho^{\text{sp}}\right]\right|^2 \\
&= \left|\mathcal{F}\left[\sqrt{\alpha}\,\rho^{\text{sp}}\right]\right|^2 + \left|\mathcal{F}\left[\sqrt{1-\alpha}\,\rho^{\text{sp}}\right]\right|^2 \\
&= \left|\mathcal{F}\left[\rho^{A}\right]\right|^2 + \left|\mathcal{F}\left[\rho^{B}\right]\right|^2
\end{aligned}
\tag{S12}
$$

where the two reconstructed frames are defined as:

$$
\begin{aligned}
\rho^{A} &= \sqrt{\alpha}\,\rho^{\text{sp}} \\
\rho^{B} &= \sqrt{1-\alpha}\,\rho^{\text{sp}}
\end{aligned}
$$

for any scalar $0 \leq \alpha \leq 1$.

Equation (S12) demonstrates that a conventional single-particle, single-color pattern can be mathematically decomposed into the sum of two diffraction intensities that are identical up to a normalization factor. The two frames ρ^A and ρ^B retrieved by Dichography are simply rescaled versions of the original solution ρ^{sp}. However, while ρ^{sp} is unique, the corresponding pair of dichographic solutions (ρ^A, ρ^B) is not. An infinite set of valid solutions exists, corresponding to every possible value of α in the interval $[0, 1]$.

This degeneracy is directly observable in Fig. S18. Multiple dichographic reconstructions were performed on the single-pulse pattern shown in Fig. S18a. The resulting reconstructions are displayed in Fig. S18b, sorted by the total intensity of the brightest frame.

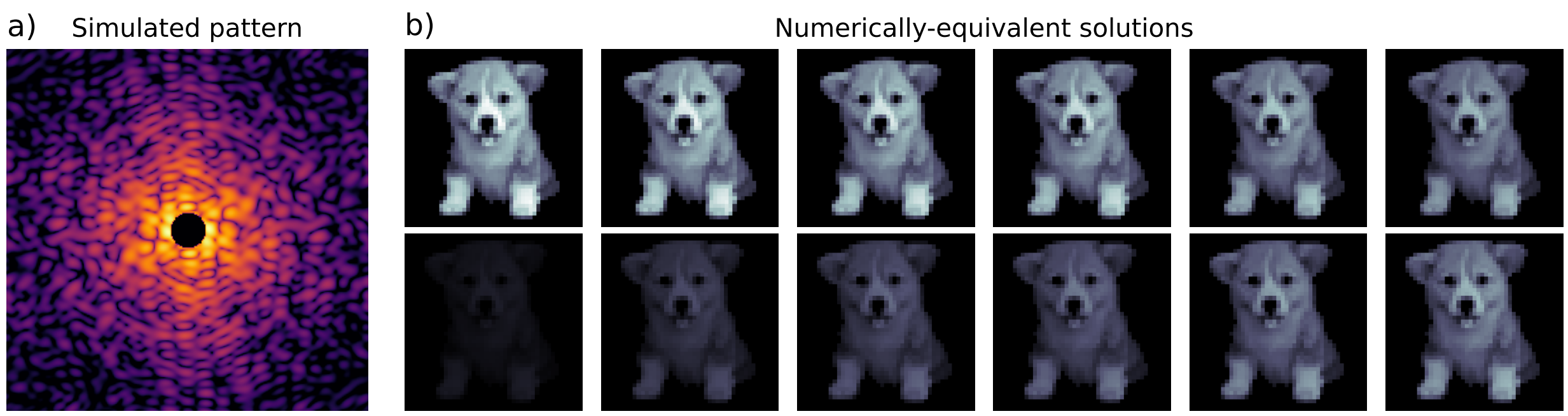

Figure S18: Degeneracy of Dichography solutions for single-pulse data. A conventional single-particle diffraction pattern is shown in a). b) reports a selection of valid dichographic solutions for the pattern in a). Since the problem admits infinite solutions of the form $\rho^A = \sqrt{\alpha}\rho^{\mathrm{sp}}$ and $\rho^B = \sqrt{1-\alpha}\rho^{\mathrm{sp}}$, the algorithm may converge to any arbitrary splitting of the intensity. The solutions are sorted from left to right by the integrated intensity of the brightest frame (top row).

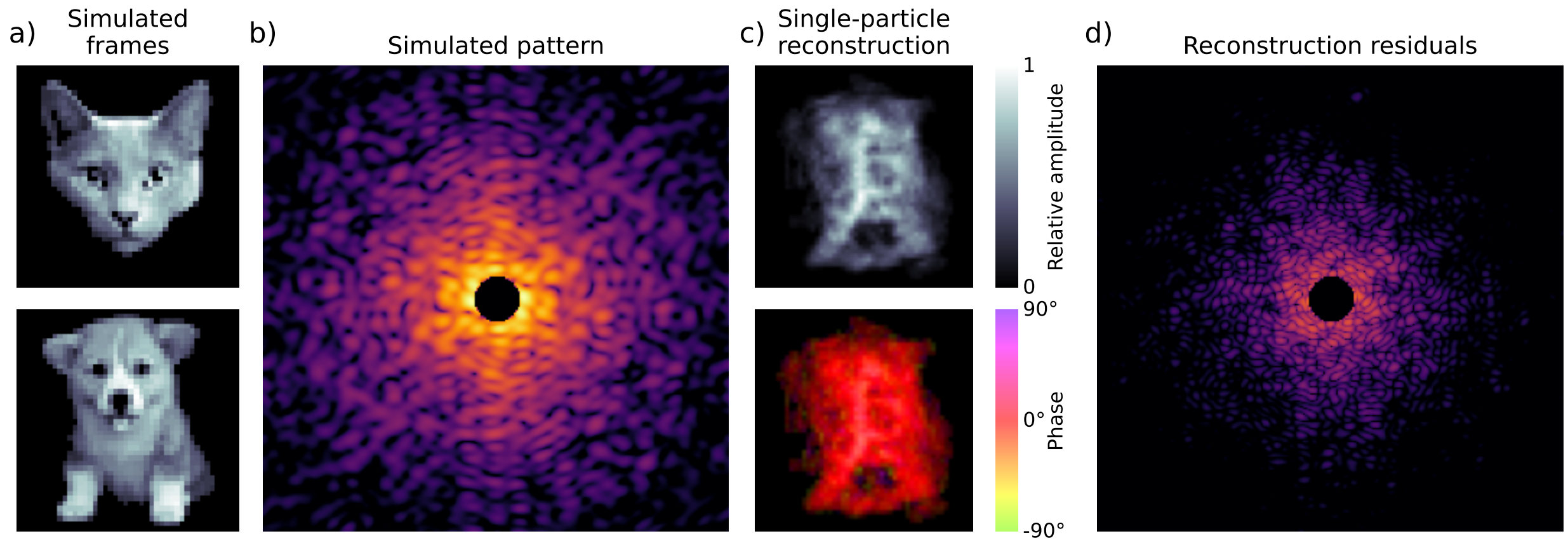

Figure S19: Conventional CDI reconstruction of a simulated noise-free dichographic pattern. The original ground-truth densities of the two frames are shown in a), while the corresponding simulated dichographic pattern is reported in b). The outcome of the conventional CDI reconstruction is shown in c), where the amplitude (top) and phase (bottom) of the complex-valued reconstructed density are reported. d) shows the residuals of the reconstruction, i.e., the difference between the reconstructed pattern and the original diffraction image. Both b) and d) are in logarithmic color scale with the same range of values, to allow for direct comparison.

S7 Dichographic patterns with conventional phase-retrieval

This section numerically investigates the feasibility of analyzing a dichographic diffraction pattern using conventional phase retrieval algorithms. As discussed in Sec. VI B 3 of the main text, a solution to the dichographic problem within the conventional CDI framework generally does not exist.

Figure S19 presents a numerical test where a standard CDI reconstruction is attempted on simulated, noise-free dichographic data. The results demonstrate that conventional phase retrieval fails to converge to a valid solution. The reconstructed density in Fig. S19c bears only a vague resemblance to the overlapping features of the two original frames. This lack of convergence is quantitatively confirmed by the residuals shown in Fig. S19d. If a valid single-particle solution existed, the residuals would ideally be zero (within numerical precision). The presence of significant structured residuals confirms that an exact solution cannot be reached.

As mentioned in Sec. VI B 3, asking whether there exists a valid conventional CDI solution corresponds to finding a spatially confined density ρ^{sp} such that:

$$\left|\tilde{\rho}^{\mathsf{sp}}(\vec{k})\right|^2 = \left|\tilde{\rho}^{A}(\vec{k})\right|^2 + \left|\tilde{\rho}^{B}(\vec{k})\right|^2 \tag{S13}$$

where $\tilde{\rho}$ denotes the Fourier Transform of the density functions.

We investigate the specific case where ρ^{sp} is restricted to be a linear combination of the two

densities, i.e., $\rho^{\text{sp}}(\vec{x}) = \alpha\rho^A(\vec{x}) + \beta\rho^B(\vec{x})$. The diffraction pattern $\left|\tilde{\rho}^{\text{sp}}(\vec{k})\right|^2$ can be expanded as:

$$\begin{aligned}\left|\tilde{\rho}^{\text{sp}}(\vec{k})\right|^2 &= \left|\alpha\tilde{\rho}^A(\vec{k}) + \beta\tilde{\rho}^B(\vec{k})\right|^2 \\ &= \left(\alpha\tilde{\rho}^A(\vec{k}) + \beta\tilde{\rho}^B(\vec{k})\right)\left(\alpha^*\tilde{\rho}^{A*}(\vec{k}) + \beta^*\tilde{\rho}^{B*}(\vec{k})\right) \\ &= |\alpha|^2\left|\tilde{\rho}^A(\vec{k})\right|^2 + |\beta|^2\left|\tilde{\rho}^B(\vec{k})\right|^2 + 2\,\text{Re}\left[\alpha\beta^*\tilde{\rho}^A(\vec{k})\tilde{\rho}^B(\vec{k})^*\right].\end{aligned} \tag{S14}$$

Eq. (S13) can thus be rewritten as a difference that must equal zero for all reciprocal coordinates $\vec{k}$:

$$\begin{aligned}0 &= \left|\tilde{\rho}^{\text{sp}}(\vec{k})\right|^2 - \left(\left|\tilde{\rho}^A(\vec{k})\right|^2 + \left|\tilde{\rho}^B(\vec{k})\right|^2\right) \\ &= (|\alpha|^2 - 1)\left|\tilde{\rho}^A(\vec{k})\right|^2 + (|\beta|^2 - 1)\left|\tilde{\rho}^B(\vec{k})\right|^2 + 2\,\text{Re}\left[\alpha\beta^*\tilde{\rho}^A(\vec{k})\tilde{\rho}^B(\vec{k})^*\right].\end{aligned} \tag{S15}$$

By definition, the two scattering densities are distinct, $\rho^A \neq \rho^B$, which implies their scattering amplitudes are different. We now consider the points in reciprocal space where the scattering amplitude is zero, referred to as *nodes*. Since the densities are different, if a node of $\tilde{\rho}^A(\vec{k})$ appears at a given coordinate, it typically does not coincide with a node of $\tilde{\rho}^B(\vec{k})$ (i.e., the latter remains non-zero).

Applying this consideration to Eq. (S15), we observe that at a node of $\tilde{\rho}^A$, the interference term vanishes. Since we assume $\left|\tilde{\rho}^B\right| \neq 0$ at this location, the equation necessitates that $|\beta| = 1$. By symmetry, evaluating the equation at a node of $\tilde{\rho}^B$ requires that $|\alpha| = 1$. Consequently, the condition $|\alpha| = |\beta| = 1$ implies that the auto-correlation terms in Eq. (S15) are identically zero. The problem thus reduces to determining whether constant phases for α and β exist such that the interference term vanishes for all $\vec{k}$:

$$\text{Re}\left[\alpha\beta^*\tilde{\rho}^A(\vec{k})\tilde{\rho}^B(\vec{k})^*\right] = \text{Re}\left[e^{i(\phi_\alpha - \phi_\beta)}\tilde{\rho}^A(\vec{k})\tilde{\rho}^B(\vec{k})^*\right] = 0. \tag{S16}$$

where $\alpha = e^{\phi_\alpha}$ and $\beta = e^{\phi_\beta}$.

This condition can be fulfilled only if $\tilde{\rho}^A(\vec{k})$ and $\tilde{\rho}^B(\vec{k})$ are "phase-locked" throughout the entire reciprocal space. Crucially, this phase-locking imposes a strict topological constraint on the fields. For general 2D objects, the zeros of the scattering intensity act as phase vortices (singularities) around which the phase winds by 2π [12]. If the phases of A and B are rigidly linked by the condition above, any topological feature in one phase must be mirrored in the other. Specifically, at a node of $\tilde{\rho}^A$, the phase ϕ^A exhibits a vortex. To maintain a constant phase difference, ϕ^B is forced to exhibit an identical vortex at the same coordinate.

However, for general distinct objects, the nodes of $\tilde{\rho}^A$ and $\tilde{\rho}^B$ do not coincide. Thus, at the location of a node of $\tilde{\rho}^A$, the amplitude of $\tilde{\rho}^B$ is typically non-zero ($\left|\tilde{\rho}^B\right| > 0$). Imposing a phase vortex at a point of non-zero amplitude implies that the complex field $\tilde{\rho}^B$ itself is discontinuous at that point. Since the Fourier Transform of a spatially confined object is analytic (and therefore single-valued and continuous everywhere), such a discontinuity is forbidden. Consequently, the phase-locking condition cannot be satisfied in the general case.

There is, however, a specific exception where this phase-locking is possible: when both fields $\tilde{\rho}^A(\vec{k})$ and $\tilde{\rho}^B(\vec{k})$ are real-valued (up to a global phase factor). This corresponds to the pathological case where both densities are *Hermitian* (point-symmetric), i.e., satisfy the condition $\rho(\vec{x})^* = \rho(-\vec{x})$. In this scenario, the interference term $\text{Re}\left[e^{i(\phi_\alpha - \phi_\beta)}\tilde{\rho}^A(\vec{k})\tilde{\rho}^B(\vec{k})^*\right]$ simplifies because the

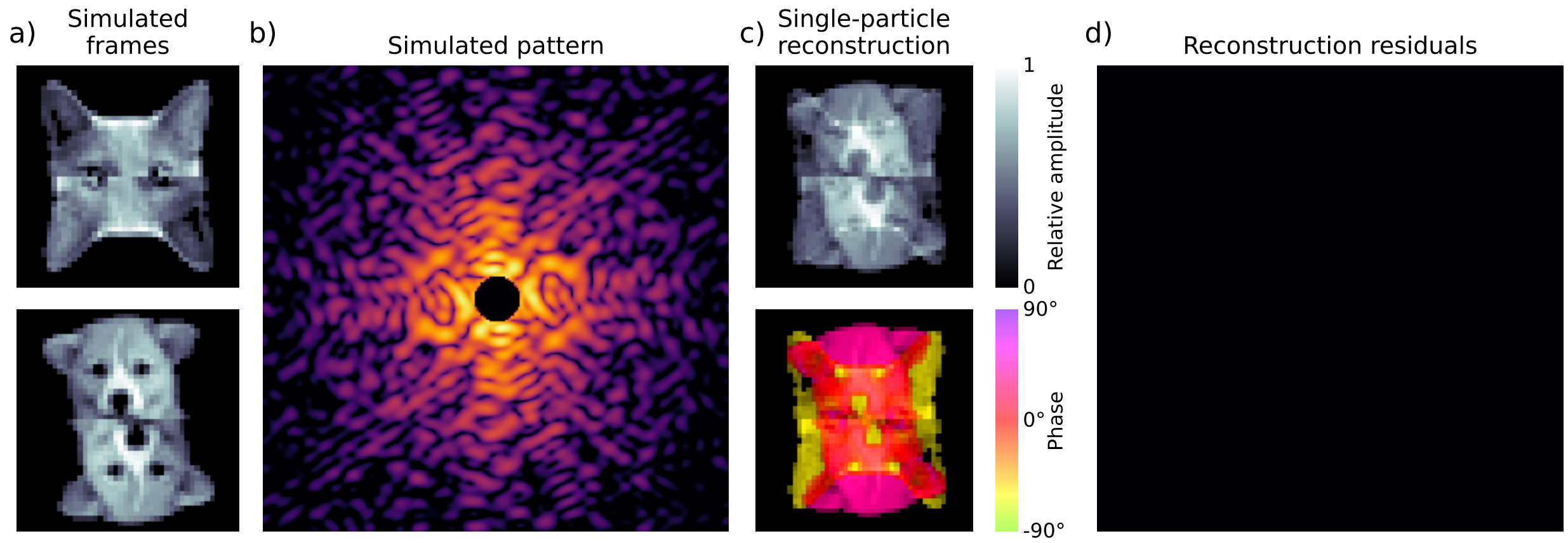

Figure S20: Conventional CDI reconstruction of simulated dichographic pattern, in the case of perfectly center-symmetric densities in both frames. The figure is internally organized as Fig. S19.

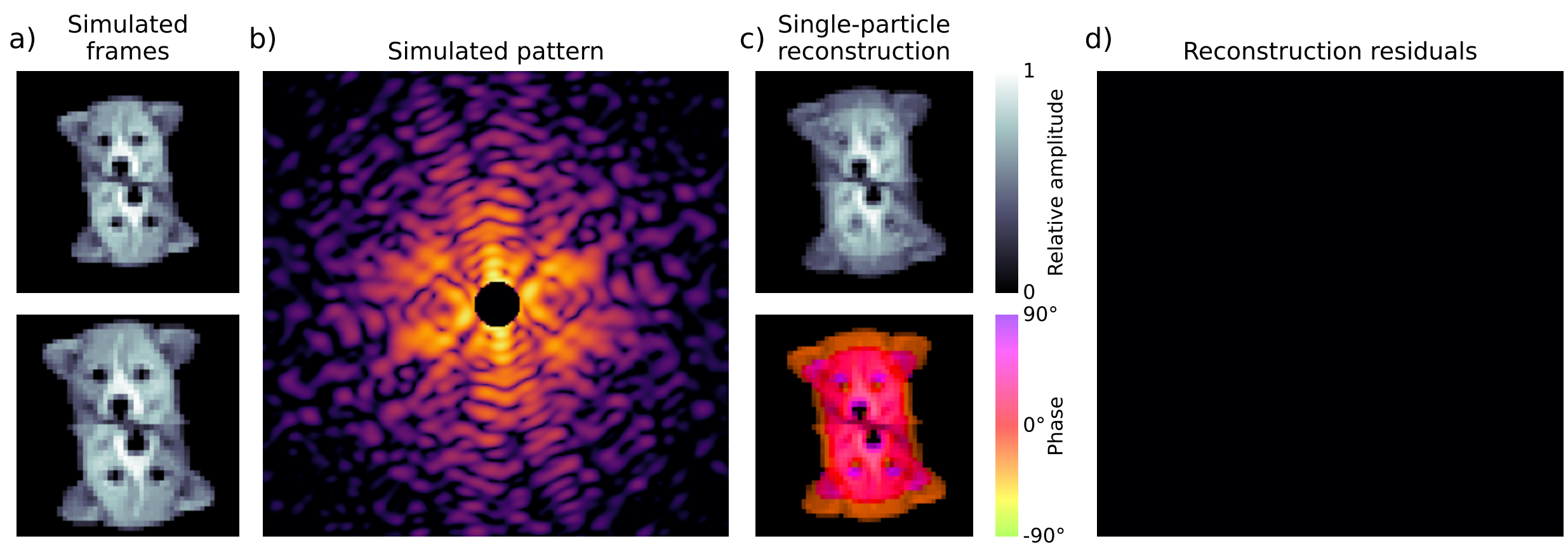

Figure S21: Conventional CDI reconstruction of simulated dichographic pattern, in the case of perfectly center-symmetric densities in both frames. The figure is internally organized as Fig. S19.

product $\tilde{\rho}^A\tilde{\rho}^{B*}$ is strictly real. Consequently, the term can be zeroed everywhere by simply assigning phases such that $\phi_\alpha - \phi_\beta = \pm\frac{\pi}{2}$.

In summary, we have investigated whether a conventional CDI solution ρ^{sp} exists for the dichographic problem as a linear combination of ρ^A and ρ^B. We conclude that such a solution exists only for the pathological case where both objects are point-symmetric. In that scenario, the solution is the linear combination of the two point-symmetric densities with coefficients $\alpha = 1$ and $\beta = \pm\mathrm{i}$ (up to a global phase factor), i.e.:

$$\rho^{\text{sp}}(\vec{x}) = \rho^A(\vec{x}) \pm \mathrm{i}\rho^B(\vec{x}). \tag{S17}$$

Fig. S20 and Fig. S21 practically show this case for the two-particle case and for the two-color case, respectively. In both cases, a solution with conventional phase retrieval can be found, which yields zero residuals. Fig. S22 underlines how the point-symmetric requirement must be present in both frames: if one of the two densities is non-symmetric, the ambiguity is resolved and the conventional reconstruction doesn't reach a solution.

Finally, Fig. S23 and Fig. S24 report the same tests of Fig. S20 and Fig. S21. Here, however,

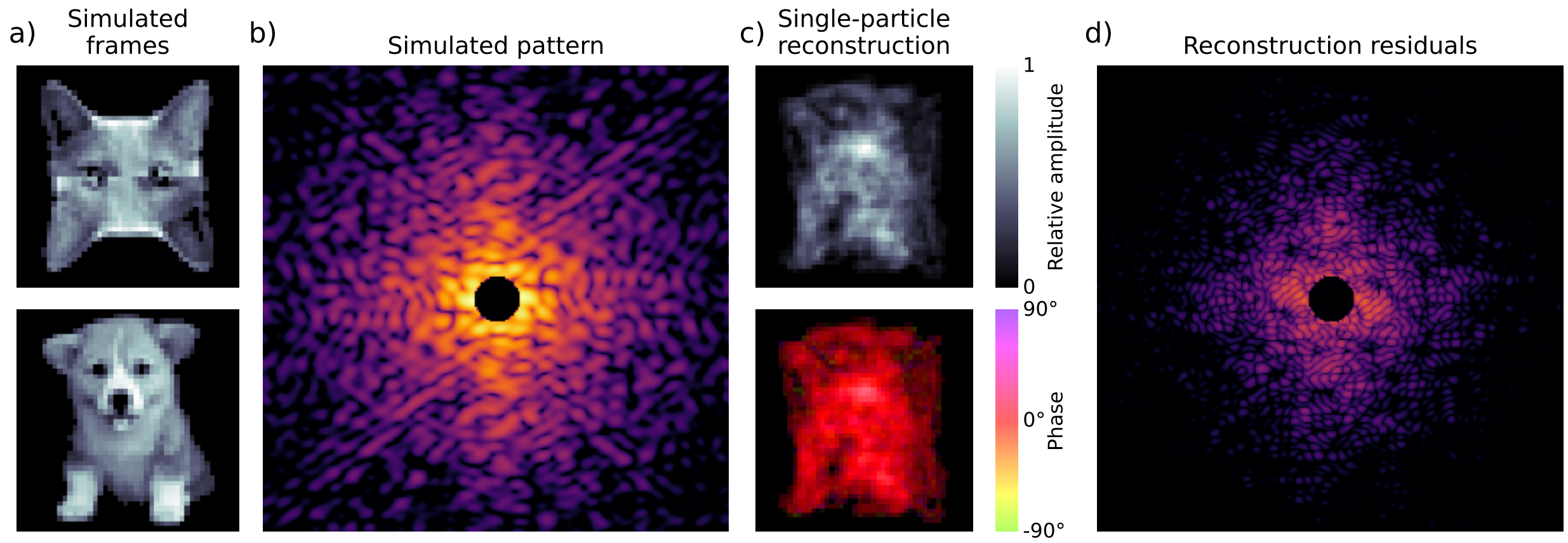

Figure S22: Conventional CDI reconstruction of simulated dichographic pattern, in the case of a perfectly center-symmetric density in one of the two frames. The figure is internally organized as Fig. S19.

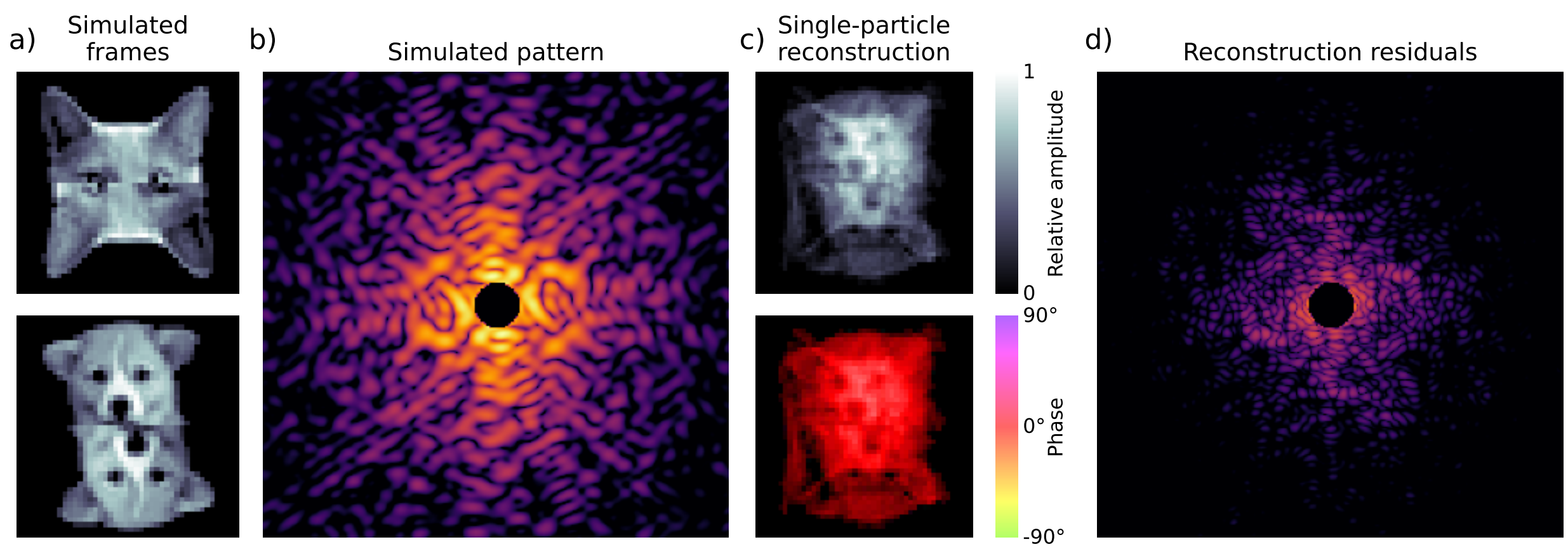

Figure S23: Conventional CDI reconstruction of simulated dichographic pattern, in the case of perfectly center-symmetric densities in both frames. The phase differences of the reconstructed density are here constrained to be smaller than $\frac{\pi}{2}$. The figure is internally organized as Fig. S19.

the phase difference between the two densities is constrained to be less than $\frac{\pi}{2}$. For such a restriction, the convergence to a conventional CDI solution is prevented and the ambiguity resolved.

S7.1 Conventional reconstructions on double-hits

The observations about conventional CDI solutions to the dichographic problem are directly verified by performing conventional phase retrieval on the experimental patterns of *double-hits*. Results are shown in Fig. S25. Here, in particular, it is worth noting the behavior of reconstructions in Fig. S25a and Fig. S25d, which corresponds to the two most point-symmetric density functions. In both cases, the CDI result resembles the linear combination of the two frames derived in Eq. S17 and numerically shown in Fig. S20 and Fig. S21.

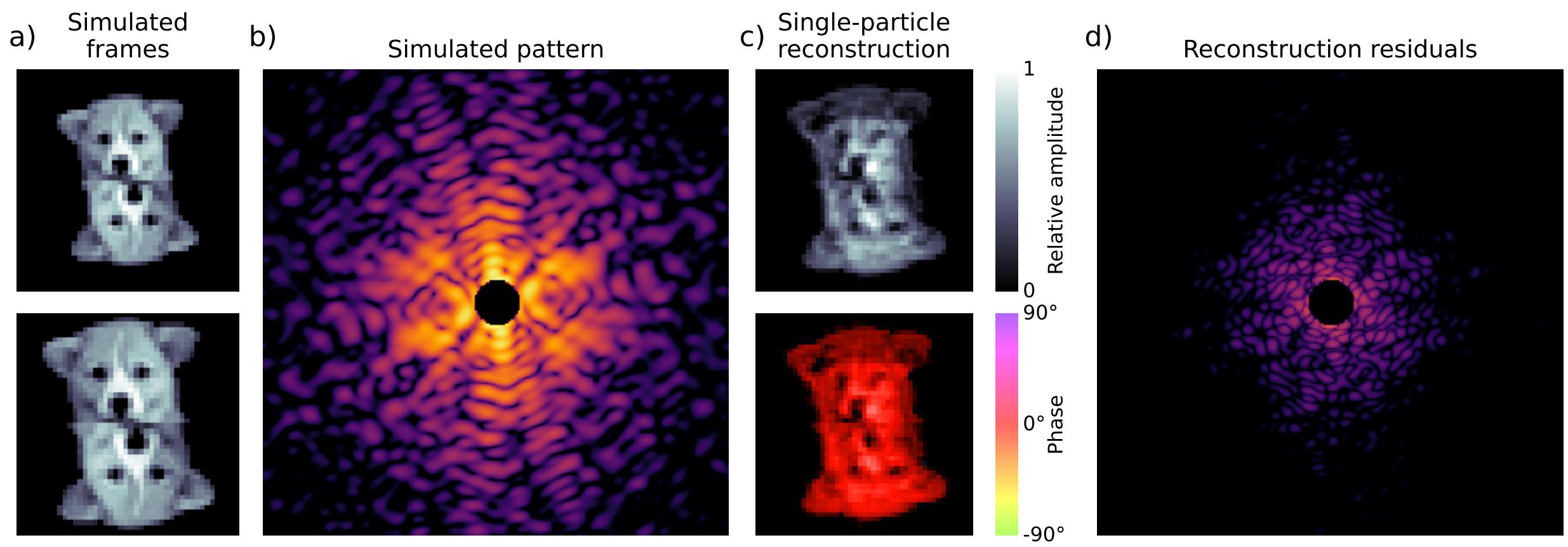

Figure S24: Conventional CDI reconstruction of simulated dichographic pattern, in the case of perfectly center-symmetric densities in both frames. The phase differences of the reconstructed density are here constrained to be smaller than $\frac{\pi}{2}$. The figure is internally organized as Fig. S19.

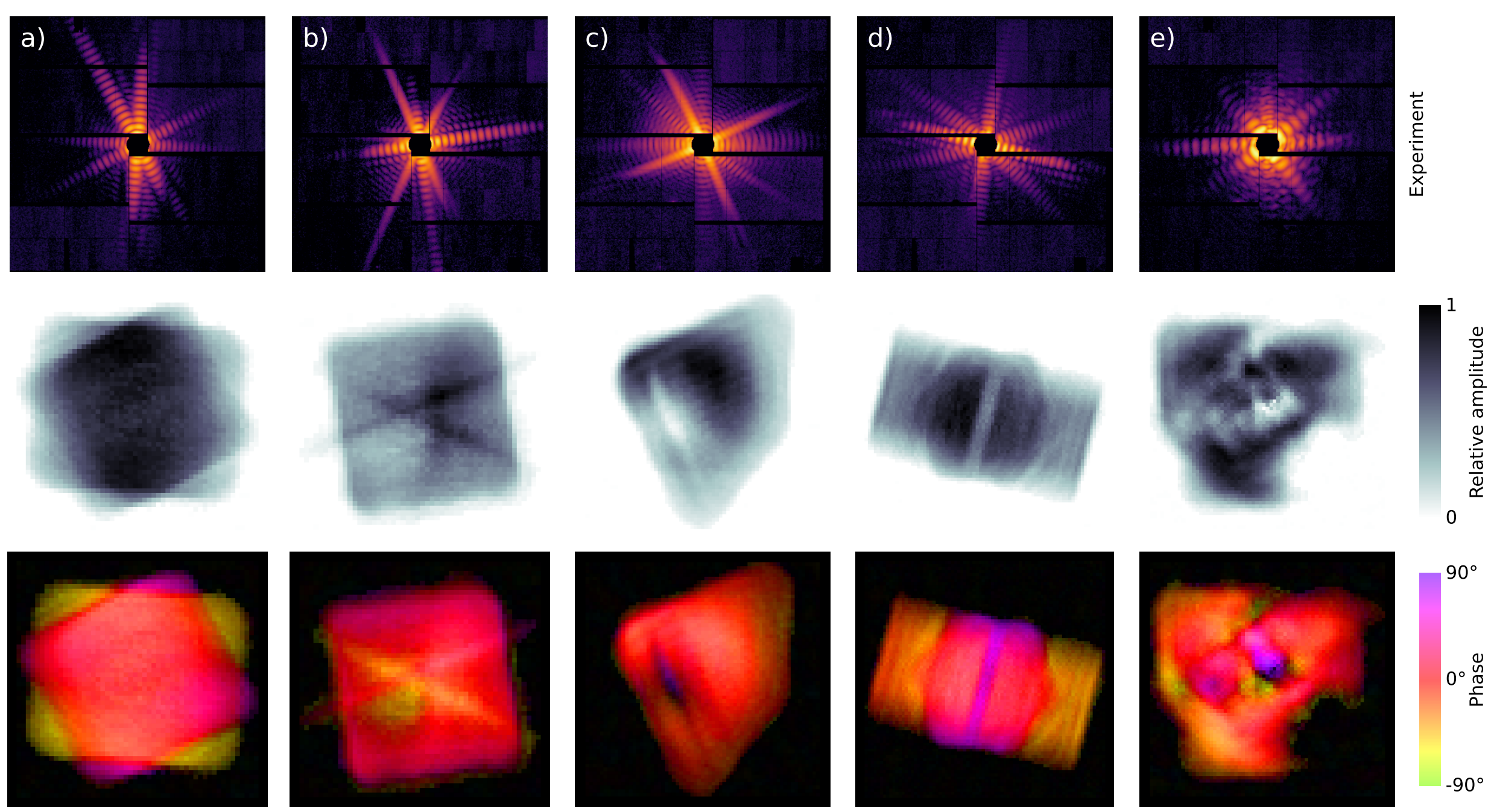

Figure S25: Conventional single-particle CDI reconstructions on the double-hits diffraction data shown in Fig. 3 and Fig. 4. Diffraction patterns are shown in the first row, in logarithmic color scale. The second and third rows show an individual reconstruction, in amplitude and phase.

S8 Experimental details

S8.1 Silver nanoparticles at SwissFEL

The experiment was performed at the Maloja endstation of SwissFEL [13, 14] (proposal 20222035). Wet-chemically grown silver nanoparticles were injected into the experimental chamber, kept under vacuum conditions, using an electrospray and a set of aerodynamic lenses [15, 16]. The electrospray disperses the liquid containing the suspended nanoparticles into sub-micron-sized droplets. The nanoparticle concentration is optimized for a single sample per droplet. The droplets flow toward the interaction region along with a buffer gas in a set of aerodynamic lenses, where the liquid evaporates.

The XFEL pulses, with a duration of $50\,\mathrm{fs}$ and a total energy of $3\,\mathrm{mJ}$, are delivered by SwissFEL at a repetition rate of $100\,\mathrm{Hz}$. The pulses are tuned to a photon energy of $1000\,\mathrm{eV}$, corresponding to a wavelength of $1.24\,\mathrm{nm}$, and intercept the nanoparticles in a $3\,\mu\mathrm{m}$ focus. These parameters correspond to a maximum intensity in the focus of approximately $10^{18}\,\mathrm{W/cm^2}$. The scattering signal is recorded by a 4-megapixel Jungfrau detector optimized for soft X-ray photon detection [8, 17]. The detector placement allows for the detection of the diffraction signal up to a scattering angle of $13.5°$.

The minimum spatial separation in the transverse direction between the two particles of the double-hit, necessary to avoid the recording of the interference term, is calculated to be $4\,\mu\mathrm{m}$. The data employed for the reconstructions was further rescaled in the post-processing phase, reducing the distance down to $1\,\mu\mathrm{m}$. Please refer to Sec. S2 of the Supplemental Material for further discussion. These quantities, and especially the $4\,\mu\mathrm{m}$ value for the raw detector data, have to be considered an upper bound. The minimum distance can be further reduced by the limited temporal and spatial coherence of the FEL pulse, assumed to be fully coherent in those calculations.

S8.2 Two-color experiment at the European XFEL

The experiment was performed at the Nano-sized Quantum Systems (NQS) endstation of the Small Quantum Systems (SQS) instrument [18, 19, 20], located on the SASE3 branch of the European XFEL [21] (proposal 2857). The XFEL was operated at a repetition rate of $10\,\mathrm{Hz}$ with an electron energy of $16\,\mathrm{GeV}$.

Two consecutive sets of XFEL undulators were tuned to $992\,\mathrm{eV}$ and $1192\,\mathrm{eV}$, respectively. A magnetic chicane located after the first undulator section delayed the arrival of the electron bunch at the second undulator, introducing a time delay of $750\,\mathrm{fs}$ between the $992\,\mathrm{eV}$ pulse and the subsequent $1192\,\mathrm{eV}$ pulse. This enables a *pump–probe* scheme in which both X-ray pulses are generated by the XFEL [22].

The two pulses, each with a nominal duration of $20\,\mathrm{fs}$, were focused to a $5\,\mu\mathrm{m}$ spot in the interaction region [20, 22], where they intercepted isolated superfluid helium nanodroplets doped with xenon atoms. The scattered light from doped helium nanodroplets, generated by both pulses, was recorded by a pnCCD detector [23] positioned at the back of the interaction region. The geometry allows capturing scattering signals up to an angle of $6.4°$ at the edge of the detector.

In this special XFEL operation mode, the intensity at the focus is significantly reduced because each pulse is produced and amplified by only half of the available undulator sections, which are tuned to different wavelengths [22]. Consequently, the energy per pulse is reduced to $0.7\,\mathrm{mJ}$, compared to more than $4\,\mathrm{mJ}$ in standard single-color operation. Furthermore, the physical separation between the undulator sections means the two collinear pulses are focused at slightly different

downstream positions. As a result, the optimal focus for two-color operation lies between these two positions, with an estimated effective focal spot size of $14\,\mu\mathrm{m}$ [6]. The combined effect of reduced pulse energy and increased focal spot size yields a nominal maximum intensity of approximately $10^{16}\,\mathrm{W/cm^2}$, about two orders of magnitude lower than that achieved in single-color mode. Data acquisition for doped droplets was limited by technical constraints and only approximately 600 hits could be recorded. From these, the final dataset was selected based on total signal brightness and, crucially, the intensity balance between the two pulses, which varies due to the spatial separation of the two foci [6]. Insights into the data selection process are reported in Sec. S2 of the SM.

S9 Adaptation of Memetic Phase Retrieval

The capability of iterative phase retrieval algorithms to reach the solution to the phase retrieval problem – i.e., to correctly reconstruct the sample density – is a key factor in CDI. These algorithms are, in fact, the virtual lenses of CDI, and the quality and reliability of the retrieved sample image are heavily dependent on their performance. For this reason, significant effort has been devoted to the development of various iterative algorithms [9]. These algorithmic approaches are well established and are routinely employed in CDI-based experimental techniques at synchrotrons, such as Bragg-CDI [24] or Ptychography [25].

Single-particle single-shot CDI performed at XFELs, however, comes with additional complications that render the algorithm's task non-trivial. For example, the central region of the diffraction pattern cannot be recorded due to the presence of a hole in the detector, necessary to prevent damage from the transmitted beam. Furthermore, the brightness of the patterns is most of the time sub-optimal and cannot be increased, as the intensity of the single XFEL pulse is already tuned to its maximum performance. These factors significantly impact the performance of iterative algorithms and render CDI at XFELs considerably more challenging.

A recent breakthrough in algorithmic capabilities comes from the development of the Memetic Phase Retrieval (MPR) approach [26, 27]. This approach takes advantage of the parallel execution of several reconstruction procedures on the same diffraction pattern. Information between these reconstructions is shared through a genetic algorithm, which augments the capabilities of conventional iterative algorithms and boosts the quality and reliability of the image reconstruction [27].

Mathematical considerations as well as systematic tests on simulated data (see Sec. S1 and Sec. S4 of the Supplemental Material) show that *Dichography* reconstructions are more demanding than conventional CDI, and that the performance of conventional iterative algorithms is further impacted. Thus, the MPR scheme has been adapted to *Dichography* resulting in a significant boost in performance, especially concerning the retrieval of the support function during the reconstruction process.

The MPR adaptation for *Dichography* is called *Equinox*. *Equinox* has been employed to obtain all imaging results presented in this manuscript, as well as those on simulated data discussed in the Supplemental Material, unless specified otherwise. It is implemented as a Python package directly derived from SPRING [27], a recently released toolset for image reconstruction in conventional single-particle single-shot CDI. The open-source implementation of *Equinox* and its documentation are accessible at `https://gitlab.ethz.ch/nux/equinox`.

Equinox retains the conceptual structure of its conventional CDI counterpart, SPRING [27]. The most significant modification, aside from the use of dichographic iterative projectors (see Sec. VI A), lies in the initialization of the starting densities. As in SPRING, the individual frames

are initialized by populating the real-space matrix with spherical density profiles of random position and size. In *Equinox*, however, the densities of the two frames are additionally renormalized to reflect unequal scattering contributions. The intensity ratio is randomly assigned within a range from 50:50 to 90:10. While these limits are arbitrary and tunable, we empirically observed that this range is sufficiently robust to reconstruct data with diverse intensity ratios without the need for case-specific fine-tuning. The algorithm can successfully retrieve the correct scattering ratio independent of the initial balancing.

Currently, *Equinox* implements the Error Reduction (ER), Hybrid Input-Output (HIO) [11], and Relaxed Averaged Alternating Reflections (RAAR) [28] algorithms. Future work includes the implementation of the Difference Map (DM) algorithm [29] adapted for the *dichographic* problem [10], which is expected to further improve the convergence properties of the solver.

S10 Retrieving the shape of helium droplets

The Droplet-CDI (DCDI) method [3] constrains the density distribution of the helium droplet during the reconstruction process, such that the phase retrieval task only involves reconstructing the density values of the inner xenon doping. The use of the DCDI method made it possible to analyze the two-color diffraction images with *Dichography*, as it greatly reduces the complexity of the imaging problem and makes it significantly more resilient to noise.

The applicability of the DCDI method to the two-color *pump-probe* data relies on two fundamental prerequisites: the ability to determine the droplet size a priori, and the structural integrity of the droplet, which must be preserved over the $750\,\mathrm{fs}$ time delay between the two XFEL pulses. Both of these conditions have been verified and demonstrated in Ref. [6], and the main findings are summarized below.

For a spherical particle, the scattered field can be calculated exactly and analytically using Mie theory, which provides solutions to Maxwell's equations [30]. The scattering signal can be fully computed with knowledge of the droplet size D, the radiation wavelength λ, the refractive index of the material $n^{\mathrm{He}}(\lambda)$ at the given photon energy, and the energy density of the light field.

For a diffraction signal from the same pure-helium droplet, composed of the incoherent sum of two different colors, Ref. [6] shows that it is possible to retrieve the size of the droplet and the intensity of the two pulses by fitting the recorded radial intensity profile as the incoherent sum of two Mie-calculated intensities, one for each color. In this procedure, the droplet size D is assumed constant between the two scattering events, while the wavelengths of the two pulses, $\lambda^A = 1.25\,\mathrm{nm}$ and $\lambda^B = 1.04\,\mathrm{nm}$, as well as the corresponding refractive indices for helium, $n^{\mathrm{He}}(\lambda^A)$ and $n^{\mathrm{He}}(\lambda^B)$ [31], are known a priori.

Furthermore, the comparison between the fitted scattering profiles and the experimental data reveals full compatibility of the recorded signal with that produced by a hard sphere, even for the signal produced by the late pulse arriving on the sample after $750\,\mathrm{fs}$. This compatibility is experimentally verified up to a spatial resolution of $19\,\mathrm{nm}$, corresponding to the momentum transfer for the $1192\,\mathrm{eV}$ photon energy at a 1.5° scattering angle. This angle has been identified as the maximum at which the signal-to-noise ratio in the recorded data is sufficiently high to provide useful information, as discussed in Ref. [6].

The observation about the integrity of helium droplets can be supported by theoretical considerations. The maximum number of electrons $n_{e,out}$ that can leave the droplet due to the interaction with the first pulse can be calculated via $n_{e,out} = (h\nu - I_p)4\pi\epsilon_0 Re^{-2}$ [32]. For a droplet with radius $R = 350\,\mathrm{nm}$ and photon energy $992\,\mathrm{eV}$, this corresponds to $n_{e,out} = 2.38 \times 10^5$ charges. This value fixes the maximum kinetic energy of a helium ion leaving the droplet and sets an upper bound to the maximum distance reached by the ion in $750\,\mathrm{fs}$, estimated as $18.6\,\mathrm{nm}$ (see Sec. S8). This upper bound is already below the $19\,\mathrm{nm}$ resolution limit of the experiment.

The physical system is thus compatible with a compact spherical helium nanodroplet for both the *pump* and the *probe* pulses, as supported by both experimental observations and theoretical considerations. Furthermore, it is possible to extract the droplet size in a precise and consistent way by fitting Mie solutions to Maxwell's equations for a signal produced by two different photon energies. For a thorough investigation of the physical system and the fitting procedure, please refer to Ref. [6]. This provides the a priori information necessary for the use of the DCDI method during the imaging process with *Dichography*.

As discussed in Sec. VI A 3, the iterative approach to *Dichography* retains the real-space operations of the conventional iterative phase retrieval algorithms for CDI. The DCDI method is, in practice, an additional real-space constraint and can be directly implemented in the algorithm workflow (see Fig. 5). The only required additional step is to properly adapt the droplet size of the

density profile to the corresponding spatial resolution for the two frames, ρ^A and ρ^B.

S10.1 Maximum number of ejected photoelectrons from a helium nanodroplet

When a helium nanodroplet is irradiated with X-ray pulses, the electrons in the system can be ejected via the photoelectric effect. The maximum number of photoelectrons $n_{\text{e,out}}$ that can leave the droplet depends on the balance between the photon energy and the Coulomb potential generated by the accumulated positive charge on the droplet. This maximum number can be estimated by equating the excess photon energy (above the ionization potential I_p) to the Coulomb potential created by the droplet charge [32]:

$$n_{\text{e,out}} = \frac{(E_{\text{ph}} - I_p)\, 4\pi\epsilon_0 R}{e^2}, \tag{S18}$$

where R is the droplet radius, E_{ph} is the photon energy, I_p is the ionization potential and e is the elementary charge. For a helium ($I_p = 24.6\,\text{eV}$) nanodroplet of size $R = 350\,\text{nm}$ illuminated by $1\,\text{keV}$ radiation, Eq. (S18) gives a maximum number of photoelectrons of $n_{\text{e,out}} = 2.4 \cdot 10^5$ emitted, before the Coulomb potential prevents further electron escape.

S10.2 Expected dynamics of ejected ions

The maximum number of ejected photoelectrons corresponds to the maximum charge accumulated in the system. Charged ions are subjected to a Coulomb repulsion and accelerated outwards. The kinetic energy $E_{\text{kin}}(r)$ of an ion at a distance $r > R$ from the center of the droplet can be calculated as:

$$E_{\text{kin}}(r) = \frac{qQ}{4\pi\epsilon_0}\left(\frac{1}{R} - \frac{1}{r}\right). \tag{S19}$$

The corresponding velocity is:

$$v(r) = \sqrt{\frac{2E_{\text{kin}}(r)}{m}} = \sqrt{\frac{qQ}{2m\pi\epsilon_0}\left(\frac{1}{R} - \frac{1}{r}\right)}, \tag{S20}$$

where m is the mass of the helium ion.
Expressing velocity as $v = \frac{dr}{dt}$, we can write:

$$\frac{dr}{dt} = \sqrt{\frac{qQ}{2m\pi\epsilon_0}\left(\frac{1}{R} - \frac{1}{r}\right)}. \tag{S21}$$

Rearranging and integrating from the droplet surface $r = R$ at $t = 0$ to $r = R + \Delta r$ at time t:

$$t = \sqrt{\frac{2m\pi\epsilon_0}{qQ}} \int_R^{R+\Delta r} \frac{dr'}{\sqrt{\frac{1}{R} - \frac{1}{r'}}}. \tag{S22}$$

This expression gives the time required for a singly charged helium ion to move a distance Δr away from the droplet surface under Coulomb repulsion. The following values are numerically calculated from Eq. (S22):

Time [fs]	Distance [nm]
50	0.09
750	18.75
1500	70.85

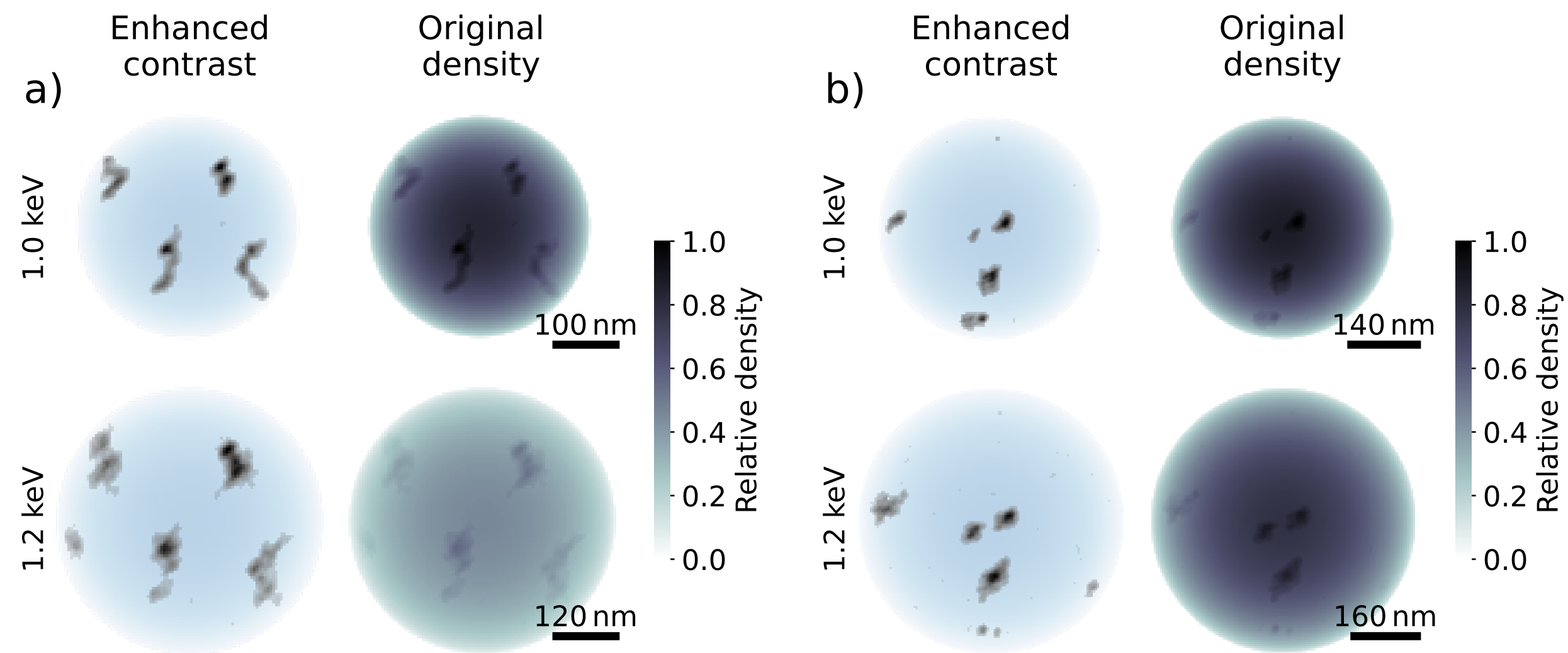

Figure S26: Two-color reconstructions of the xenon-doped helium nanodroplets shown in Fig. 2, here reported with the original sampling. The figure presents the same data as shown in Fig. 2. Each two-frame reconstruction is reported twice. On the left, the densities are reported with enhanced contrast, similarly to Fig. 2, to highlight the inner xenon structures. On the right, the original scattering density is reported. In this second case, the color scale is shared between the two frames to also report the imbalance between the scattering contributions.

S10.3 Representation of two-color reconstructions

The spatial extension of a single pixel in the reconstruction corresponds to the half-period resolution [33]. In the small-angle approximation [34], the spatial extension of a single reconstruction pixel Δ can be calculated as:

$$\Delta = \frac{\lambda}{2\theta_{\text{max}}} \tag{S23}$$

where λ is the radiation wavelength and θ_{max} is the angle measured from the center of the detector to its side edge. For a two-color reconstruction with *Dichography*, the two frames correspond to two views of the same sample obtained with different colors. As such, the corresponding pixel size Δ, i.e., the sampling in real space, differs. In particular, the values for the $1.0\,\mathrm{keV}$ ($\lambda^A = 1.25\,\mathrm{nm}$) pulse and the $1.2\,\mathrm{keV}$ ($\lambda^B = 1.04\,\mathrm{nm}$) pulse can be calculated via Eq. (S23) as $\Delta^{1.0\,\mathrm{keV}} = 5.57\,\mathrm{nm}$ and $\Delta^{1.2\,\mathrm{keV}} = 4.64\,\mathrm{nm}$.

As a consequence, when reconstructing a sample of size D, the $1.0\,\mathrm{keV}$ reconstruction will have an extent of $D/\Delta^{1.0\,\mathrm{keV}}$ pixels, while the $1.2\,\mathrm{keV}$ reconstruction will extend over $D/\Delta^{1.2\,\mathrm{keV}}$ pixels. To better highlight this aspect, we report in Fig. S26 the same two-color reconstructions as shown in Fig. 2, where the two retrieved frames are not scaled to match the spatial extension; i.e., they are cropped from the reconstructed matrices ρ^A and ρ^B with the same pixel dimensions. On the one hand, this representation helps the reader visualize the lower resolution of the $1.0\,\mathrm{keV}$ pulse.

On the other hand, it highlights that the appearance of two similar structures in the two frames cannot derive from a *ghosting* effect. In fact, the arising of a reconstruction feature in the wrong frame wouldn't match the proper spatial resolution, since the two structures differ in pixel extent. In addition, Fig. S26 reports the two scattering densities without the enhancement of the contrast for the xenon structures and with a common color scale between the two frames. The reconstructed densities are reported with the correct scaling of the helium droplet $\rho_{\bigcirc}$ and the xenon doping ρ_{χ}, i.e., $\rho = \rho_{\bigcirc} + \rho_{\chi}$. This allows the reader to better appreciate the relatively low contrast of the xenon filaments within the droplets, and thus better evaluate the noise visible in the reconstructions shown in enhanced contrast in Fig. 2. Furthermore, it is worth noting that the scattering density ρ, outcome of the reconstruction, is proportional to the actual electronic density ρ_e and the field amplitude $\sqrt{I}$, i.e., $\rho \propto \sqrt{I} \cdot \rho_e$. The visibly fainter $1.2\,\mathrm{keV}$ reconstruction in Fig. S26a comes indeed from the lower intensity contribution of the $1.2\,\mathrm{keV}$ pulse in the scattering, which corresponds to a weaker $1.2\,\mathrm{keV}$ field in the specific position of the imaged droplet within the two focal regions.

Supplementary References

[1] philippe, "Background black with Russian Blue cat." Wallpapers.com `https://wallpapers.com/wallpapers/background-black-with-russian-blue-cat-b5r01rz6wgpm0ui5.html`, 2026. Available under a free-use license with attribution.

[2] 1383, "A cute and playful Corgi pup!." Wallpapers.com `https://wallpapers.com/background/corgi-background-rhd99fob9x6u9st6.html`, 2026. Available under a free-use license with attribution.

[3] R. M. P. Tanyag, C. Bernando, C. F. Jones, C. Bacellar, K. R. Ferguson, D. Anielski, R. Boll, S. Carron, J. P. Cryan, L. Englert, *et al.*, "Communication: X-ray coherent diffractive imaging by immersion in nanodroplets," *Structural Dynamics*, vol. 2, no. 5, 2015.

[4] A. Ulmer, A. Heilrath, B. Senfftleben, S. M. O'Connell-Lopez, B. Kruse, L. Seiffert, K. Kolatzki, B. Langbehn, A. Hoffmann, T. M. Baumann, *et al.*, "Generation of large vortex-free superfluid helium nanodroplets," *Physical review letters*, vol. 131, no. 7, p. 076002, 2023.

[5] R. M. P. Tanyag, C. F. Jones, C. Bernando, S. M. O. O'Connell, D. Verma, and A. F. Vilesov, "CHAPTER 8. Experiments with Large Superfluid Helium Nanodroplets," in *Theoretical and Computational Chemistry Series* (O. Dulieu and A. Osterwalder, eds.), pp. 389–443, Cambridge: Royal Society of Chemistry, 2017.

[6] L. Hecht, Y. Ovcharenko, A. Ø. Lægdsmand, B. Bastian, T. M. Baumann, A. Colombo, S. De, A. D. Fanis, S. Dold, T. Fennel, R. Hartmann, K. Kolatzki, S. Krishnan, B. Kruse, A. C. Laforge, B. Langbehn, S. Mandal, T. Mazza, C. Medina, C. Peltz, T. Pfeifer, B. Senfftleben, K. Sishodia, F. Stienkemeier, R. M. P. Tanyag, P. Tümmler, S. Usenko, A. Heidenreich, M. Meyer, D. Rupp, and M. Mudrich, "Two-color x-ray coherent diffraction imaging of helium nanodroplets," 2025.

[7] H. N. Chapman, A. Barty, S. Marchesini, A. Noy, S. P. Hau-Riege, C. Cui, M. R. Howells, R. Rosen, H. He, J. C. Spence, *et al.*, "High-resolution ab initio three-dimensional x-ray diffraction microscopy," *Journal of the Optical society of America A*, vol. 23, no. 5, pp. 1179–1200, 2006.

[8] A. Mozzanica, M. Andrä, R. Barten, A. Bergamaschi, S. Chiriotti, M. Brückner, R. Dinapoli, E. Fröjdh, D. Greiffenberg, F. Leonarski, *et al.*, "The jungfrau detector for applications at

synchrotron light sources and xfels," *Synchrotron Radiation News*, vol. 31, no. 6, pp. 16–20, 2018.

[9] S. Marchesini, "Invited article: A unified evaluation of iterative projection algorithms for phase retrieval," *Rev. Sci. Instrum.*, vol. 78, no. 1, p. 011301, 2007.

[10] R. P. Millane and J. P. Chen, "Phase retrieval for multiple objects from their averaged diffraction," *Journal of the Optical Society of America A*, vol. 32, no. 7, pp. 1317–1329, 2015.

[11] J. R. Fienup, "Phase retrieval algorithms: a comparison," *Appl. Opt.*, vol. 21, no. 15, pp. 2758–2769, 1982.

[12] M. Soskin and M. Vasnetsov, "Singular optics," in *Progress in optics*, vol. 42, pp. 219–276, Elsevier, 2001.

[13] Z. Sun, A. Al Haddad, S. Augustin, G. Knopp, J. Knurr, K. Schnorr, and C. Bostedt, "Ultrafast single-particle imaging with intense x-ray pulses," *Chimia*, vol. 76, no. 6, pp. 529–529, 2022.

[14] PSI, "Swissfel maloja experimental station." `https://www.psi.ch/en/swissfel/maloja`, Aug 2025.

[15] J. Bielecki, M. F. Hantke, B. J. Daurer, H. K. Reddy, D. Hasse, D. S. Larsson, L. H. Gunn, M. Svenda, A. Munke, J. A. Sellberg, *et al.*, "Electrospray sample injection for single-particle imaging with x-ray lasers," *Science Advances*, vol. 5, no. 5, p. eaav8801, 2019.

[16] M. F. Hantke, J. Bielecki, O. Kulyk, D. Westphal, D. S. Larsson, M. Svenda, H. K. Reddy, R. A. Kirian, J. Andreasson, J. Hajdu, *et al.*, "Rayleigh-scattering microscopy for tracking and sizing nanoparticles in focused aerosol beams," *IUCrJ*, vol. 5, no. 6, pp. 673–680, 2018.

[17] V. Hinger, A. al Haddad, R. Barten, A. Bergamaschi, M. Brückner, M. Carulla, S. Chiriotti-Alvarez, R. Dinapoli, S. Ebner, E. Fröjdh, *et al.*, "Advancing the jungfrau detector toward low-energy x-ray applications," *Journal of Instrumentation*, vol. 17, no. 09, p. C09027, 2022.

[18] SQS, "Small Quantum Systems Instrument." `https://www.xfel.eu/facility/instruments/sqs/index_eng.html`, Aug 2025.

[19] T. Tschentscher, C. Bressler, J. Grünert, A. Madsen, A. P. Mancuso, M. Meyer, A. Scherz, H. Sinn, and U. Zastrau, "Photon beam transport and scientific instruments at the european xfel," *Applied Sciences*, vol. 7, no. 6, p. 592, 2017.

[20] T. Mazza, T. M. Baumann, R. Boll, A. De Fanis, P. Grychtol, M. Ilchen, J. Montaño, V. Music, Y. Ovcharenko, N. Rennhack, D. E. Rivas, A. Rörig, P. Schmidt, S. Usenko, P. Ziołkowski, D. La Civita, M. Vannoni, H. Sinn, B. Keitel, E. Plönjes, U. F. Jastrow, A. Sorokin, K. Tiedtke, K. Mann, B. Schäfer, N. Breckwoldt, S.-K. Son, and M. Meyer, "The beam transport system for the Small Quantum Systems instrument at the European XFEL: Optical layout and first commissioning results," *Journal of Synchrotron Radiation*, vol. 30, pp. 457–467, Mar. 2023.

[21] W. Decking, S. Abeghyan, P. Abramian, A. Abramsky, A. Aguirre, C. Albrecht, P. Alou, M. Altarelli, P. Altmann, K. Amyan, *et al.*, "A mhz-repetition-rate hard x-ray free-electron laser driven by a superconducting linear accelerator," *Nat. Photonics*, vol. 14, no. 6, pp. 391–397, 2020.

[22] S. Serkez, W. Decking, L. Froehlich, N. Gerasimova, J. Grünert, M. Guetg, M. Huttula, S. Karabekyan, A. Koch, V. Kocharyan, *et al.*, "Opportunities for two-color experiments in the soft x-ray regime at the european xfel," *Applied Sciences*, vol. 10, no. 8, p. 2728, 2020.

[23] M. Kuster, K. Ahmed, K.-E. Ballak, C. Danilevski, M. Ekmedžić, B. Fernandes, P. Gessler, R. Hartmann, S. Hauf, P. Holl, M. Meyer, J. Montaño, A. Münnich, Y. Ovcharenko, N. Rennhack, T. Rüter, D. Rupp, D. Schlosser, K. Setoodehnia, R. Schmitt, L. Strüder, R. M. P. Tanyag, A. Ulmer, and H. Yousef, "The 1-megapixel pnccd detector for the small quantum systems instrument at the european xfel: system and operation aspects," *J. Synchrotron Radiat.*, vol. 28, no. 2, pp. 576–587, 2021.

[24] V. Favre-Nicolin, F. Mastropietro, J. Eymery, D. Camacho, Y. Niquet, B. Borg, M. Messing, L.-E. Wernersson, R. Algra, E. Bakkers, *et al.*, "Analysis of strain and stacking faults in single nanowires using bragg coherent diffraction imaging," *New Journal of Physics*, vol. 12, no. 3, p. 035013, 2010.

[25] M. Guizar-Sicairos and P. Thibault, "Ptychography: A solution to the phase problem," *Physics Today*, vol. 74, no. 9, pp. 42–48, 2021.

[26] A. Colombo, D. E. Galli, L. De Caro, F. Scattarella, and E. Carlino, "Facing the phase problem in coherent diffractive imaging via memetic algorithms," *Sci. Rep.*, vol. 7, no. 1, p. 42236, 2017.

[27] A. Colombo, M. Sauppe, A. A. Haddad, K. Ayyer, M. Babayan, R. Boll, R. Dagar, S. Dold, T. Fennel, L. Hecht, G. Knopp, K. Kolatzki, B. Langbehn, F. R. N. C. Maia, A. Mall, P. Mazumder, T. Mazza, Y. Ovcharenko, I. C. Polat, D. Raiser, J. C. Schäfer-Zimmermann, K. Schnorr, M. L. Schubert, A. Sehati, J. A. Sellberg, B. Senfftleben, Z. Shen, Z. Sun, P. H. W. Svensson, P. Tümmler, S. Usenko, C. F. Ussling, O. Veteläinen, S. Wächter, N. Walsh, A. V. Weitnauer, T. You, M. Zuod, M. Meyer, C. Bostedt, D. E. Galli, M. Patanen, and D. Rupp, "SPRING, an effective and reliable framework for image reconstruction in single-particle Coherent Diffraction Imaging," *npj Computational Materials*, vol. 11, no. 1, p. 265, 2025.

[28] D. R. Luke, "Relaxed averaged alternating reflections for diffraction imaging," *Inverse problems*, vol. 21, no. 1, pp. 37–50, 2005.

[29] V. Elser, "Phase retrieval by iterated projections," *Journal of the Optical Society of America A*, vol. 20, no. 1, pp. 40–55, 2003.

[30] G. Mie, "Beiträge zur Optik trüber Medien, speziell kolloidaler Metallösungen," *Annalen der Physik*, 1908.

[31] B. L. Henke, E. M. Gullikson, and J. C. Davis, "X-ray interactions: photoabsorption, scattering, transmission, and reflection at e= 50-30,000 ev, z= 1-92," *Atomic data and nuclear data tables*, vol. 54, no. 2, pp. 181–342, 1993.

[32] C. Bostedt, H. Thomas, M. Hoener, T. Möller, U. Saalmann, I. Georgescu, C. Gnodtke, and J.-M. Rost, "Fast electrons from multi-electron dynamics in xenon clusters induced by inner-shell ionization," *New J. Phys.*, vol. 12, p. 083004, Aug. 2010.

[33] A. Colombo and D. Rupp, "Imaging clusters and their dynamics with single-shot coherent diffraction," 2023.

[34] R. A. Kirian and H. N. Chapman, “Imaging of objects by coherent diffraction of x-ray free-electron laser pulses,” in *Synchrotron Light Sources and Free-Electron Lasers*, pp. 1135–1195, Springer International Publishing, 2016.
