# Supplementary material for: Dichography: two-frame ultrafast imaging from a single diffraction pattern
Source: Nat Commun. 2026 Jun 24;17:7944. doi: 10.1038/s41467-026-74442-w (PMC13448170; doi:10.1038/s41467-026-74442-w)
Supplement: Supplementary file 2 [file 41467_2026_74442_MOESM2_ESM.pdf]

nature portfolio

Peer Review File

Dichography: Two-frame Ultrafast Imaging from a Single Diffraction Pattern

Corresponding Author: Dr Alessandro Colombo

This file contains all reviewer reports in order by version, followed by all author rebuttals in order by version.

Version 0:

Reviewer comments:

Reviewer #1

(Remarks to the Author)
In the present manuscript, the authors introduce a technique that they term dichography which is able to solve the phase problem in X-ray imaging experiments for two incoherently summed diffraction patterns. They motivate and demonstrate the technique mainly for an experiment from the European XFEL, where they recorded diffraction patterns illuminated by two X-ray pulses 750fs delayed of two different colors. The main motivation for the technique development is the ability to reconstruct images from those double pulses with the goal to record "movies" of nanomatter (with two frames). They additionally apply the same method to retrieve images from diffraction patterns that probed two objects in the beam at a distance that is too large to be resolved by the detector, thus again creating an incoherent superposition.

The manuscript is very well written and provides an interesting approach to a complex problem. The data analysis is sound and well-described.
Nevertheless, I have concerns about the applicability of the method and would like to hear the comments by the authors and potentially additions to the manuscript before the work can be published:
1. Applying the method to double hits from the SwissFEL experiment is an elegant way of analysing the data, but left me wondering how such double hits were identified. How would the image look like, if such a double hit would have been directly analysed with the phase-retrieval algorithm without assuming a double hit? How can the authors ensure that the data actually belongs to double hits and not to single or even triple (incoherent) hits? How unique is the solution if the actual number of incoherently summed particles is unknown?
The authors have actually recorded data at higher resolutuon than they used for the reconstruction. I suspect that they used the additional information from the higher resolution diffraction pattern (two-particle interference fringes) to determine that two particles intersected the beam and they artificially removed that information by downsampling the recorded patterns. Thus the authors used external information to actually solve the phase problem (by enforcing that two particles were in the beam and not one or three). This leaves doubts about the potential of this method.
I feel these points should be discussed in a revised version of the manuscript.

2. Appying the method to the double pulses from EuXFEL, the authors also chose to ignore information: in ref. 35 of the manuscript, they show that the detector can actually (mostly) determine the photon energy of (many of) the detected photons and thus uniquely assign the detected photons to either of the two pulses. In the present manuscript, the authors chose to ignore this assignment and instead develop a method to retrieve the images anyway. This may constitute an interesting algorithmic development, but leaves questions towards the necessity of this development effort. Especially at higher photon energies, the absolute energy separation between two-color pulses grows and makes it even easier to assign photon counts to one of the two pulses.

Besides these major overarching comments, I wonder what the actual fraction of useful images from EuXFEL was. The authors claim that only a few number of shots could be analysed. It would be interesting to determine the current applicability of the method by getting a number of what fraction of shots was usable or unusable with a reason assignment (like too few photons, too large imbalance between both pulses, droplet not hit or the like).

Further, I wonder if the authors are aware of the early work in DOI: 10.1038/nphoton.2010.287, where another approach to record two time-separated images with X-rays is shown.

Lastly, some editorial issues that occurred to me:
l. 366 and 368 should refer to Figure 3.
l. 405 contains a dobule word.
l. 861 and l. 867 are duplicate versions.
l. 1087 refers to the wrong experiment.

In conclusion, the manuscript might be suitable for publication after the authors have discussed in more detail why they chose to ignore part of the data and how that relates to the motivation of this work. At present, the algorithm seems to solve a problem that the authors artificially created. I am happy to review a revision of the manuscript.

Reviewer #2

(Remarks to the Author)
The paper by Hecht et al. reports Dichography, a method that enables the separation and reconstruction of individual images from two independent, overlapping diffraction patterns. Conventional Coherent Diffraction Imaging (CDI) experiments rely on several constraints, among which k space isolation is essential. The manuscript is noteworthy for demonstrating that phase retrieval can be successfully applied to two or more samples that are not isolated in k-space but are nevertheless spatially separated sufficiently to avoid mutual interference. The technique shows strong potential for expansion, not only by enabling simultaneous imaging of multiple samples, but also by enabling X-ray pump–probe imaging and by allowing experiments to retain data that would otherwise be disregarded for failing isolation criteria. Despite these strengths, several important issues should be addressed. I list questions and suggestions below.

1. Dichography appears to be a powerful technique capable of reconstructing two images from a single diffraction pattern. When CDI was first introduced, the reliability of its reconstructions (before uniqueness was fully established) was typically demonstrated by comparing results with those from independent imaging techniques such as SEM images. Although the convergence properties of iterative phase-retrieval algorithms help manage noise, they do not, by themselves, guarantee that the recovered image is correct.

In the case of the droplet experiment, it seems experimentally difficult to image the same sample using an independent method. This limitation means that Dichography, as applied here, cannot be directly validated by an external experiment, which is a significant drawback. To address this, we suggest implementing a self-validation approach. If Dichography can produce two images of identical structure but differing sampling conditions when the two-color delay time is set to zero, this would provide strong internal evidence that the method functions as claimed. Have the authors performed such a zero-delay two-color measurement but chosen not to include it in the manuscript? If not, I believe that additional experiments are essential. Simulations alone are not sufficient to establish the validity of the technique under these conditions.

2. Distinguishing ghosting artifacts is a critical issue for the proper application of Dichography. The authors state that the result in Fig. 4 cannot be attributed to ghosting, since the structures appear at the same spatial positions but differ in size. However, the simulations in Fig. S8 suggest that this criterion may not be reliable. As the photon number decreases, the reconstruction quality degrades substantially, to the point where it becomes unclear whether the recovered features correspond to the true object. For instance, in Fig. S8(g) at 1.0 keV, distinct features appear in regions where no structure exists in the ground-truth object. In the simulation, one can identify which features belong to the true image and which are artifacts or ghosting, because the original image is known. In an actual experiment, however, the true image is not known. Under such circumstances, how can one determine whether a reconstructed feature is real or a ghosting artifact?

3. Due to the characteristics of XFEL, both the pulse intensity and beam position can fluctuate significantly from shot to shot. This means that even if the intensity of each color in the two-color mode is monitored, the ratio of the diffracted intensities may not scale proportionally. In the manuscript, it appears that the scattering ratio α is determined algorithmically rather than directly from measured intensity ratios. It needs an explanation for the validity. If Fig. S13(d) through Fig. S15(d) correspond to this algorithmic determination of α, it would be helpful to indicate this explicitly on the y-axis to avoid confusion for readers.

4. In line 528, the authors mention the spatial resolution. Because CDI relies on iterative phase retrieval, the resolution is determined by how accurately the phase is recovered at high spatial frequencies. Therefore, the resolution should be evaluated, e.g., by phase retrieval transfer functon(PRTF), to support any discussion of spatial resolution.

5. It is not well-described what the initial conditions are used for in Dichography. In standard phase retrieval algorithms, it is common to begin with a random initial phase to initiate the reconstruction. In Dichography, however, not only the object phases but also the modulation term ϕ^M must be retrieved. This implies that ϕ^M requires an initial setting as well. Do the authors initialize ϕ^M with a uniform value (e.g., 1 at all points), or do the authors start from a random distribution? Please clarify what initial condition was used and the rationale behind this choice.

6. In the last paragraph on page 4 of the Supplemental Material, it is stated that small samples are more resilient to noise because of their higher oversampling degree. However, smaller samples usually produce weaker scattering signals, which lowers the signal-to-noise ratio (SNR) and typically makes successful reconstruction more challenging. By contrast, larger samples scatter more strongly because of their larger cross-sections, resulting in higher SNR. For this reason, the current explanation appears inconsistent with the expected physical behavior. We suggest revising this paragraph to address this discrepancy.

Reviewer #3

(Remarks to the Author)
I co-reviewed this manuscript with one of the reviewers who provided the listed reports. This is part of the Nature Communications initiative to facilitate training in peer review and to provide appropriate recognition for Early Career Researchers who co-review manuscripts.

Version 1:

Reviewer comments:

Reviewer #1

(Remarks to the Author)
The authors have substantially revised and extended their previous submission and have in my opinion addressed all the points that were raised by the other referees as well as in my comment. I believe that the paper is ready for publication in the present form.

Reviewer #2

(Remarks to the Author)
The revised manuscript has been substantially improved and now addresses the main technical concerns raised during review. Although a true zero-delay experiment was not experimentally feasible, the authors provide a reasonable justification that the 50 fs delay effectively serves as a practical validation point for this system, and the updated Fig. 2 reflects this point appropriately. The added theoretical support also strengthens the methodological basis of the work.

The discussion of ghosting artifacts and the uniqueness of the solution is now clearer and more convincing. In particular, the revised explanation of the mathematical origin of ghosting improves the reader's understanding of the limitations and reliability of the reconstructions. The additional explanation regarding the algorithmic determination of the scattering ratio is also appropriate and resolves the earlier ambiguity.

The inclusion of the PRTF analysis is an important improvement, and the reported resolution appears sufficient to support the authors' claims. The newly added clarification of the initialization procedure in Dichography is also necessary and helpful. Finally, the revised explanation concerning the relationship between sample size, SNR, and missing low-q information resolves the previous inconsistency well.

Overall, the manuscript has been significantly strengthened by the revision and is now suitable for publication.

Reviewer #3

(Remarks to the Author)

Open Access This Peer Review File is licensed under a Creative Commons Attribution 4.0 International License, which permits use, sharing, adaptation, distribution and reproduction in any medium or format, as long as you give appropriate credit to the original author(s) and the source, provide a link to the Creative Commons license, and indicate if changes were made.
In cases where reviewers are anonymous, credit should be given to 'Anonymous Referee' and the source.
The images or other third party material in this Peer Review File are included in the article's Creative Commons license, unless indicated otherwise in a credit line to the material. If material is not included in the article's Creative Commons license and your intended use is not permitted by statutory regulation or exceeds the permitted use, you will need to obtain permission directly from the copyright holder.
To view a copy of this license, visit https://creativecommons.org/licenses/by/4.0/

RESPONSE TO REVIEWERS' COMMENTS

Dear Reviewers,
We express our gratitude to the referees for their thorough reviews. Their comments regarding the uniqueness of the solution, the data selection process, and the relevance of the presented data prompted a significant amount of additional work. This effort proved pivotal for a deeper understanding of the problem and a more rigorous presentation of the results in our revised manuscript. We are convinced that the quality of the manuscript has been greatly improved and its scientific impact augmented.

We also wish to highlight that, during the review process, we became aware of a previous work by Millane and Chen (J. Opt. Soc. Am. A 32, 1317, 2015), which established a theoretical framework for an analogous imaging problem (ensemble imaging beyond crystallography), including a formal demonstration of solution uniqueness. This finding solidifies the theoretical foundation of our approach, allowing us to focus on the key value of our current work: the practical experimental realization of two-frame imaging. Additionally, we exchanged correspondence with prof. Millane, whose additional feedback helped in further strengthening our updated Methods section. We acknowledge his input in the revised manuscript.

A concise overview of the key improvements in the revised manuscript is listed below:

Sec. I (The Dichographic problem): We now explicitly mention that the problem has been investigated theoretically and that the existence of a solution has been demonstrated for idealized cases.
Sec. II (Results): The two-color imaging results are now presented first to better highlight their primary significance in this work.
Sec. IIA ("Two-color Coherent Diffraction Imaging of xenon-doped helium nanodroplets"): Addressing the referees' comments, we developed a more rigorous method to identify usable diffraction patterns in the two-color dataset. This allowed us to identify another diffraction image of sufficient quality at 50 fs time delay, which yields a convincing reconstruction. This replaces the second 750 fs reconstruction shown in the first version. Furthermore, algorithmic refinements allowed for a more robust reconstruction of the 750 fs pattern despite its low SNR.
Sec. IV (Outlook): We clarify the connection with Ref. [38] (formerly [35]), explaining why pixel-wise separation can aid Dichography but cannot replace it. We also briefly discuss outlooks for 3D imaging and multi-color experiments.
Sec. V.A.1 (Iterative phase retrieval): Figure 5 has been updated to explicitly show that the support functions are independently retrieved along the reconstruction process.
Sec. V.A.2 (Constraining experimental data): The content has been adjusted to avoid misunderstandings regarding the complexity of the Dichographic problem.
Sec. V.B.1: The section "Oversampling requirements for Dichography" has been replaced by "Constraint requirements for Dichography". The previous discussion on oversampling degree is replaced by a more rigorous treatment in terms of the constraint ratio, following Millane and Chen (2015).
Sec. V.B.2: New section "Constraint ratio for Droplet Coherent Diffraction Imaging". We introduce a modified constraint ratio that accounts for the peculiarities of DCDI, enabling a quantitative evaluation of the two-color results within the same framework.

Sec. V.B.3: New section “Non-existence of a conventional CDI solution”. Following the reviewers' suggestion, we provide theoretical arguments demonstrating that, in general, dichographic data does not possess a valid conventional CDI solution.

Sec. V.B.4 (Uniqueness of the solution): This section has been completely rewritten to focus on the inherent “rotational” ambiguity of the solution and how the support constraint resolves it. We explicitly link "ghosting" artifacts to this rotational ambiguity.

Sec. V.C (Adaptation of Memetic Phase Retrieval): We extended this section to provide previously missing details on density initialization, as requested by the referees.

Sec. V.F (Representation of two-color reconstructions): We updated the figures to include both two-color reconstructions. Data is also presented in "raw" format (without enhancement of xenon structures) to clarify the retrieved signal against the helium background.

Sec. S2 (SM): New section “Statistics and data selection of the two-color experiment”. We present a quantitative evaluation that explains why suitable patterns for Dichography are rare in the recorded data due to the specific setup of this experiment.

Sec. S2.1 (SM): New section “Spatial resolution of two-color reconstructions”. As requested, we provide a resolution estimate via the Phase Retrieval Transfer Function (PRTF) and discuss its interpretation in the context of Dichography.

Sec. S6 (SM): **New section “Conventional single-particle patterns with Dichography”**, investigating the behavior of the algorithm when applied to conventional single-pulse data.

Sec. S7 (SM): New section “Dichographic patterns with conventional phase-retrieval”, where the theoretical conclusions of Sec. V.B.3 are numerically investigated on both simulated and experimental data.

In addition to these main points, we have implemented local text improvements in different sections of the manuscript. Attached you find a pdf version with all changes highlighted in blue. In the following, we address the queries raised by the referees.

Reviewer #1:

In the present manuscript, the authors introduce a technique that they term dichography which is able to solve the phase problem in X-ray imaging experiments for two incoherently summed diffraction patterns. They motivate and demonstrate the technique mainly for an experiment from the European XFEL, where they recorded diffraction patterns illuminated by two X-ray pulses 750fs delayed of two different colors. The main motivation for the technique development is the ability to reconstruct images from those double pulses with the goal to record "movies" of nanomatter (with two frames). They additionally apply the same method to retrieve images from diffraction patterns that probed two objects in the beam at a distance that is too large to be resolved by the detector, thus again creating an incoherent superposition.
The manuscript is very well written and provides an interesting approach to a complex problem. The data analysis is sound and well-described.
Nevertheless, I have concerns about the applicability of the method and would like to hear the comments by the authors and potentially additions to the manuscript before the work can be published:

We thank the reviewer for their positive assessment of our work.

1. Applying the method to double hits from the SwissFEL experiment is an elegant way of analysing the data, but left me wondering how such double hits were identified. How would the image look like, if such a double hit would have been directly analysed with the phase-retrieval algorithm without assuming a double hit? How can the authors ensure that the data actually belongs to double hits and not to single or even triple (incoherent) hits? How unique is the solution if the actual number of incoherently summed particles is unknown?
The authors have actually recorded data at higher resolution than they used for the reconstruction. I suspect that they used the additional information from the higher resolution diffraction pattern (two-particle interference fringes) to determine that two particles intersected the beam and they artificially removed that information by downsampling the recorded patterns. Thus the authors used external information to actually solve the phase problem (by enforcing that two particles were in the beam and not one or three). This leaves doubts about the potential of this method.
I feel these points should be discussed in a revised version of the manuscript.

The referee raises three important points: the way the double-hits are identified, the significance of the double-hit diffraction analysis, and the connection between Dichography and conventional CDI.

Identification of double hits. Regarding the identification of double hits and distinguishing them from triple or quadruple hits, we wish to stress two important aspects. First, the probability of these multiple (n>2) hits is extremely low. With a typical overall hit-rate of 1% in single-particle CDI at FELs (often dictated by experimental conditions such as the target density produced by an

injector, but in general also a necessary requirement to stay in “single-particle” mode, i.e. ensuring predominantly a single particle in the focus), a triple-hit event occurs only once every few hours of data acquisition. Second, as the main scope of the method is the two-color application, we focus on the case where at most two incoherent signals are present. However, regarding the specific identification of double-hit patterns (versus single hits) were manually identified. Interference in the original data was not a deciding factor; the diffraction images shown in the paper do not even show signatures of interference in the raw data (as discussed in Section S2 of the SM). While a "trained eye" can recognize double-hit patterns with relatively high accuracy, the manual selection served only as a pre-selection. The ultimate confirmation is the imaging reconstruction itself, as discussed below. Furthermore, we note that this identification process is usually done in this way in conventional single-particle CDI experiments, where multiple hits can be identified by the lack of convergence of the reconstruction process, and, in that case, discarded. This is theoretically justified, as explained in the new Sec. V.B.3 (“Non-existence of a conventional CDI solution”).

Significance of Double-Hit Analysis. We concur with the referee that the application of Dichography to the image reconstruction of double-hits is an elegant way to analyze the data. Indeed, we included this double-hit analysis to demonstrate the method on "solid" experimental patterns with high signal-to-noise ratio. Double-hits themselves are rare events (see above), comprising (in the best case) only a few percent of the total recorded data. We agree that the ability to reconstruct images from double hits has limited relevance in itself compared to the two-color application, which is the focal point of this work. However, the double-hit dataset serves as a crucial proof of concept, allowing us to test Dichography on experimental data that is not affected by the shot noise inherent to the real two-color data.
We have decided to change the order of presented results to focus more on the two-color data and clarified this idea of an additional benchmark-case by implementing the following change in the Introduction:
"While the analysis of these double-hit patterns is of limited scope regarding the physical implications, it serves as a crucial benchmark dataset. Indeed, it allows us to show and investigate the performance of the method on experimental data for optimal diffraction conditions, i.e. not affected by the limited performance of FELs in two-color operation mode, and for diverse structural properties of the samples."

Dichography vs. Conventional CDI. The referee asks: What is the outcome of Dichography when fed with a conventional single-particle pattern? And vice versa? These questions touch the essence of the method. We have investigated this problem both numerically and analytically, adding dedicated sections to the manuscript and Supplemental Material (SM).
Dichography on Single-Particle Patterns. As discussed in the new Section S5 of the SM ("Conventional single-particle patterns with Dichography"), there is an infinity of valid solutions when Dichography is fed a single-particle pattern. These correspond to two replicas of the same image, scaled such that their sum matches the data. The algorithm reveals this ambiguity by converging to different linear combinations for different random initializations (see Fig. S18). This failure to converge to a unique solution is a robust indication that the data does not contain a two-color/two-particle signal.

CDI on Dichographic Patterns. We have added a new section to the main manuscript, "Non-existence of a conventional CDI solution," which provides theoretical arguments based on Hayes' theorem on polynomial factorization. We prove that, in general, a single-particle solution to the Dichographic problem cannot exist. While "pathological" cases exist (e.g., perfectly centro-symmetric densities), we show in the new Section S7 of the SM how these can be resolved numerically. We also show that reconstructing the double-hit patterns with conventional CDI (see Fig. S25) results in phase fluctuations incompatible with a physical single particle.

2. Appying the method to the double pulses from EuXFEL, the authors also chose to ignore information: in ref. 35 of the manuscript, they show that the detector can actually (mostly) determine the photon energy of (many of) the detected photons and thus uniquely assign the detected photons to either of the two pulses. In the present manuscript, the authors chose to ignore this assignment and instead develop a method to retrieve the images anyway. This may constitute an interesting algorithmic development, but leaves questions towards the necessity of this development effort. Especially at higher photon energies, the absolute energy separation between two-color pulses grows and makes it even easier to assign photon counts to one of the two pulses.

The referee correctly connects the present manuscript with Ref. 35 (now Ref. 38, also under review at Nature Communications). While the link between the two manuscripts is strong, the works are fundamentally complementary.

Ref. 35 demonstrates how the photon signal can be color-separated experimentally, but this relies on the fundamental condition that the average number of photon events per pixel stays below 0.1 (sparse regime). Therefore, the two techniques are not exclusive but complementary:
Low scattering angles (High Brightness). Pixel-based separation fails. Dichography is essential here to disentangle the strong, overlapping signals that define the overall shape and features of the object.
High scattering angles (Low Brightness). Dichography struggles due to shot noise. Here, the experimental color separation (Ref. 35) becomes powerful because the photon density is low enough for single-photon counting.

We agree that combining these methods is a high priority, and we are already working on adapting the algorithmic flow to include partial information from color separation. However, optimizing this combination requires assessing performance as a function of pattern brightness and ratio, which is a complex parameter space suitable for a separate, dedicated study. The aim of the current work is to communicate the fundamental finding that two incoherent signals from unknown scattering densities can be fully disentangled mathematically, serving as a pillar for these future developments. To clarify this distinction in the text, we have modified the Outlook section of the manuscript to explicitly state:
"This is achieved by exploiting the sensitivity of the detector to the photon's energy combined with advanced analysis techniques, and can be employed only in the regions of the scattering signal where photon events are sufficiently rare [Ref. 38], typically at high scattering angles."

Besides these major overarching comments, I wonder what the actual fraction of useful images from EuXFEL was. The authors claim that only a few number of shots could be analysed. It would be interesting to determine the current applicability of the method by getting a number of what fraction of shots was usable or unusable with a reason assignment (like too few photons, too large imbalance between both pulses, droplet not hit or the like).

The data presented in this manuscript and in Ref. 35 (now Ref. 38) originate from the same experiment (Proposal 2857 at European XFEL). The experimental plan contained two different targets: pure helium nanodroplets and xenon-doped droplets. While a substantial dataset was acquired for pure droplets, the acquisition for doped droplets was unfortunately curtailed by a failure in the cooling system of the superconducting magnets leading to a premature end of the beamtime. Consequently, we were able to record only a limited dataset for the xenon-doped helium droplets (approx. 660 hits, one to two orders of magnitude less than a typical CDI experiment).

From this limited pool, the selection of "useful" images for Dichography is governed by a specific physical constraint inherent to this two-color setup. As detailed in Ref. 35, the foci of the two colors are generated at different positions in the undulators section, therefore their source points are several meters apart from each other. This leads to collinear beams, but as they are focused by the same X-ray optics, their foci are also spatially separated along the beam axis by a few centimeters. As a consequence, the relative intensity of the two pulses experienced by a droplet varies significantly as it depends on the droplet's exact position within the interaction region. As such, the contributions of the individual colors can't be determined by an external measurement of the color ratios (e.g., gas-monitor detectors or integration of single-shot spectra) but it has to be extracted from the patterns alone. A fundamental requirement for Dichography, especially at low diffraction intensities, is that the two pulses contribute comparable signal levels. Shots where one pulse dominates are effectively single-pulse patterns and are not suitable for two-color reconstruction. Due to the peculiar properties of the beams in the interaction region, it turns out that patterns where the intensity contribution between the two signals is balanced represent a "rare event".

We have addressed this in detail in the new Section S2 of the Supplemental Material ("Statistics and data selection of the two-color experiment"). There, we describe a rigorous two-step selection process:

Initial Filtering. We first select patterns based on total brightness and the ratio of retrieved photon energies (derived from the DCDI fitting procedure), isolating shots that match the nominal experimental parameters on the ratio between the photon energies. This first step allows us to select only those shots where structural information from both colors is present in the diffraction.

Refined Selection. We then evaluate the "normalized brightness" (information density per pixel relative to particle size) against the retrieved intensity balance of the two colors. As shown in Fig. S10, only a small fraction of hits fall into the optimal regime where both high information content and a near 1:1 intensity balance are achieved.

Crucially, the rigorous application of these selection criteria, prompted by the referee's query, enabled us to identify a new high-quality diffraction pattern at a 50 fs delay. The reconstruction from this pattern is now shown in Fig. 2a of the main manuscript and represents a new key result, significantly improving the quality and impact of the experimental demonstration.

Further, I wonder if the authors are aware of the early work in DOI: 10.1038/nphoton.2010.287, where another approach to record two time-separated images with X-rays is shown.

We agree that this work represents a milestone in two-color ultrafast imaging. Indeed, it is a fundamental example of achieving time-resolved imaging by spatially separating information on the detector via an holographic approach.
We now explicitly cite this work in the Introduction among the pioneering successful realizations of two-pulse X-ray imaging:
"First successful realizations achieved this by ... or different regions of the detector through holographic approaches [32]."

Lastly, some editorial issues that occurred to me:
l. 366 and 368 should refer to Figure 3.
l. 405 contains a double word.
l. 861 and l. 867 are duplicate versions.
l. 1087 refers to the wrong experiment.

We implemented the corrections, and we thank the referee for such a careful reading of the paper.

In conclusion, the manuscript might be suitable for publication after the authors have discussed in more detail why they chose to ignore part of the data and how that relates to the motivation of this work. At present, the algorithm seems to solve a problem that the authors artificially created. I am happy to review a revision of the manuscript.

We believe that the impression that "the algorithm seems to solve a problem that the authors artificially created" may have resulted from our initial manuscript's heavy reliance on the double-hit benchmark, and a lack of clarity about the fundamental limitations of detectors.
We wish to clarify that for the primary motivation of this work, two-color ultrafast imaging, the problem is not artificially created but physically fundamental. As detailed in our response to Point 2 and the revised Outlook, in the low-angle diffraction regime essential for imaging, the photon flux is too high for the detector to resolve individual photon energies. Therefore, "ignoring" the information from pixel-based separation of the signal is not a choice, but a necessity imposed by the physics of the detector in the high-brightness regime (which inherently contains most of the structural information). Dichography solves the phase problem exactly where experimental pixel-based color separation fails.

As detailed above, we have revised the manuscript to center the narrative on the two-color results, relegating the double-hit analysis to a supporting benchmark role. The possibility of retrieving sample images, and thus a two-frame movie, from the sum of incoherent scattering signals addresses a long-standing open question in the field. We believe the successful demonstration of Dichography will significantly augment the utility of two-color capabilities at FEL facilities worldwide, unlocking experimental possibilities that were previously inaccessible.

Reviewer #2:

The paper by Hecht et al. reports Dichography, a method that enables the separation and reconstruction of individual images from two independent, overlapping diffraction patterns. Conventional Coherent Diffraction Imaging (CDI) experiments rely on several constraints, among which k space isolation is essential. The manuscript is noteworthy for demonstrating that phase retrieval can be successfully applied to two or more samples that are not isolated in k-space but are nevertheless spatially separated sufficiently to avoid mutual interference. The technique shows strong potential for expansion, not only by enabling simultaneous imaging of multiple samples, but also by enabling X-ray pump–probe imaging and by allowing experiments to retain data that would otherwise be disregarded for failing isolation criteria.
Despite these strengths, several important issues should be addressed. I list questions and suggestions below.

We thank the referee for the overall positive comment on the work, and for having recognized the impact of the method and its applicability to diverse experimental schemes with a particular focus on X-ray pump X-ray probe imaging at FELs, which is the main highlight of the work.

1. Dichography appears to be a powerful technique capable of reconstructing two images from a single diffraction pattern. When CDI was first introduced, the reliability of its reconstructions (before uniqueness was fully established) was typically demonstrated by comparing results with those from independent imaging techniques such as SEM images. Although the convergence properties of iterative phase-retrieval algorithms help manage noise, they do not, by themselves, guarantee that the recovered image is correct.

In the case of the droplet experiment, it seems experimentally difficult to image the same sample using an independent method. This limitation means that Dichography, as applied here, cannot be directly validated by an external experiment, which is a significant drawback. To address this, we suggest implementing a self-validation approach. If Dichography can produce two images of identical structure but differing sampling conditions when the two-color delay time is set to zero, this would provide strong internal evidence that the method functions as claimed. Have the authors performed such a zero-delay two-color measurement but chosen not to include it in the manuscript? If not, I believe that additional experiments are essential.

Simulations alone are not sufficient to establish the validity of the technique under these conditions.

A zero-delay measurement, as suggested by the referee, where the two retrieved images contain identical information, serving as an ideal "self-validation" control, wasn't experimentally feasible at EuXFEL at the time of the experiment. The magnetic chicane used to delay the pulses has a minimum achievable delay of 50 fs. Still, as reported in Ref. [35] (now Ref. [38]) , molecular dynamics simulations show that X-ray-induced structural changes in the sample at the irradiation

level reached by the experiment only take place on lengthscales smaller than the achieved resolution of ~20nm, even at the long time delay of 750 fs. Thus, the 50 fs time-delay reconstruction can be considered, for what concerns the method validation, as meaningful as a time zero measurement for this specific sample. To better clarify this aspect, we have added the following statements in the discussion on the two-color reconstructions:
"*Thus, these results suggest that, at the irradiation level reached in this experiment, significant structural damage of the xenon filaments only happens at longer timescales. This observation, which holds up to the achieved resolution, is supported by molecular dynamics simulations [38].*"

Furthermore, we address the request for validation through a comprehensive set of experimental, theoretical, and methodological improvements:
Minimum-Delay Reconstruction. We performed a rigorous refinement of our data selection criteria (see response to Reviewer #1). This allowed us to identify a high-quality two-color diffraction pattern recorded at the minimum achievable delay of 50 fs. This result is now presented as the first experimental example in the revised manuscript (Fig. 2a). While the experimental boundary conditions prevented a true zero-delay measurement, this reconstruction demonstrates the success of the method at the closest experimentally achievable limit.
Experimental Validation. We believe the double-hit analysis serves effectively as the validation control. We apply Dichography to real experimental data from solid silver nanoparticles, i.e. stable, well-known objects. The successful reconstruction of these independent particles from the incoherent sum of their diffraction patterns provides "experimental proof" that the algorithm functions correctly on real X-ray data, without relying on simulations.
Theoretical Validation. Regarding the theoretical foundation, we were not aware of the work by Millane and Chen (2015) at the time of our first submission. Discovering this work now has provided a strong, independent theoretical validation of our approach. Millane and Chen demonstrate mathematically that, given favorable constraint ratios, the reconstruction of multiple objects from averaged diffraction intensities has a unique solution. This confirms that the problem we are solving is mathematically well-posed.
Their work, however, is purely theoretical and relies on the assumption that the object supports (shapes) are known a priori. In this regard, our work took a fundamental step further by demonstrating that reconstruction is possible in real experimental conditions (i.e., lack of data at the center of the detector and noise) and without assuming any prior knowledge of the sample shape (implementing dynamic support retrieval). Furthermore, we provide the first practical experimental demonstrations of this concept.
We have now cited Millane and Chen (2015) in the revised manuscript to contextualize our findings within this established theoretical framework, heavily restructuring the content in the Methods section and adding information in the Supplemental Material.

2. Distinguishing ghosting artifacts is a critical issue for the proper application of Dichography. The authors state that the result in Fig. 4 cannot be attributed to ghosting, since the structures appear at the same spatial positions but differ in size. However, the simulations in Fig. S8 suggest that this criterion may not be reliable. As the photon number decreases, the reconstruction quality degrades substantially, to the point where it becomes unclear whether

the recovered features correspond to the true object. For instance, in Fig. S8(g) at 1.0 keV, distinct features appear in regions where no structure exists in the ground-truth object. In the simulation, one can identify which features belong to the true image and which are artifacts or ghosting, because the original image is known. In an actual experiment, however, the true image is not known. Under such circumstances, how can one determine whether a reconstructed feature is real or a ghosting artifact?

We fully agree that in the experimental two-color reconstructions, the signal-to-noise ratio is currently not high enough to definitively exclude the presence of ghosting artifacts. However, as noted in our previous response, the primary goal of this work is to establish the feasibility of the framework, even if current FEL machine parameters for two-color modes remain at the limit of what is needed for high-resolution imaging.
To address the referee's query regarding how to distinguish real features from artifacts, we have made several key additions to the manuscript.
Mathematical Origin of Ghosting. We have completely revised the section "Uniqueness of the solution" in the main manuscript to provide a more rigorous explanation. We demonstrate that there exists, in principle, an infinite family of solutions that can satisfy the incoherent sum of intensities. These solutions are related by a rotation matrix that linearly mixes the true densities. While the support constraint (spatial isolation) and positivity typically resolve this ambiguity in the "clean signal" limit, shot noise in experimental data allows the algorithm to converge to solutions that are slightly "rotated", i.e. mixed. These mixtures manifest as ghosting.
Simulations. As pointed out by the referee, artifacts indeed appear in the low-photon simulations of Fig. S8. Rather than undermining the method, we believe this highlights the importance of these simulations; they allow us to characterize the "signature" of ghosting for a given experimental geometry. By observing how artifacts behave under controlled noise levels, we have a ruler for evaluating the reliability of experimental features.

The identification of artifacts is a long-standing challenge in all forms of single-particle imaging. For instance, conventional CDI is frequently plagued by the "twin-image" problem, where non-centrosymmetric features are reconstructed as symmetric due to stagnation in a local optimum. Similarly to the ghosting effect for Dichography, the twin-image problem is a possible artifact that has to be carefully taken into account when evaluating a reconstruction result, without hindering the importance and overall validity of the technique.

3. Due to the characteristics of XFEL, both the pulse intensity and beam position can fluctuate significantly from shot to shot. This means that even if the intensity of each color in the two-color mode is monitored, the ratio of the diffracted intensities may not scale proportionally. In the manuscript, it appears that the scattering ratio α is determined algorithmically rather than directly from measured intensity ratios. It needs an explanation for the validity. If Fig. S13(d) through Fig. S15(d) correspond to this algorithmic determination of α, it would be helpful to indicate this explicitly on the y-axis to avoid confusion for readers.

Indeed, the scattering ratio alpha is determined algorithmically and, as discussed above, the experimental situation is even more stochastic than the referee describes. Beyond the inherent pulse-intensity jitter and beam position fluctuations, the primary source of uncertainty is the longitudinal focus offset. Because the two colors are generated at different positions in the undulator hall, their foci are separated in space and the single particles are intercepted by widely varying intensity ratios, depending on their position along the XFEL axis. Consequently, any external instrumental monitoring of the pulse energy ratio cannot predict the diffracted intensity ratio. For these reasons, Dichography was specifically designed to treat the relative intensity as a free parameter.

The validity of this approach is supported by the fact that the relative intensity of the two frames is encoded in the interference-free sum of the intensities. Much like conventional CDI, where the overall density normalization (the missing central peak) is naturally retrieved during the phasing process, Dichography restores the relative weight of the two signals by minimizing the error between the observed total intensity and the sum of the two reconstructed frames. As shown in Figs. S4–S7 of the Supplemental Material, the algorithm accurately retrieves the ground-truth images across a wide range of intensity ratios without any a-priori constraints.

Regarding the specific figures mentioned, we must clarify that panel (d) in Figs. S13–S15 (now S15-S17) does not represent the intensity ratio. Instead, it plots the reconstruction error ratio, showing how the total error is distributed between the two density estimates as the algorithm iterates. This provides insight into the convergence behavior but is not a direct measure of the physical intensity ratio. To avoid confusion, we have added a clarification in the Supplemental Material stating:

"It is worth underlining that the error ratio in S13d, while related to the relative signal strength, cannot be trivially linked to the overall intensity ratio of the two frames; rather, it shows how the two frames contribute differently to the overall error as a function of the iterations."

4. In line 528, the authors mention the spatial resolution. Because CDI relies on iterative phase retrieval, the resolution is determined by how accurately the phase is recovered at high spatial frequencies. Therefore, the resolution should be evaluated, e.g., by phase retrieval transfer functon (PRTF), to support any discussion of spatial resolution.

As requested, we have performed a Phase Retrieval Transfer Function (PRTF) analysis on the experimental two-color reconstructions shown in the new Fig. 2 (updated two-color results). The analysis is now included in the revised Supplemental Material (Section S2.1 and Fig. S11).

The PRTF analysis yields a half-period resolution of around 20 nm for the two reconstructions presented in Fig. 2, respectively. Given the low signal-to-noise ratio inherent in the current two-color FEL data, we prefer to report the resolution at the conservative PRTF = 0.5 threshold, rather than the less stringent 1/e criterion often used in literature (which yields slightly better resolutions of around 15 nm, respectively, as further shown in the SM).

We also clarify a fundamental limitation of PRTF in the context of Dichography. Because the PRTF is calculated against the total experimental diffracted intensity, it primarily reflects the resolution of the brightest contribution and cannot independently disentangle the resolutions of the two frames. In particular, we added the following observation when presenting the PRTF analysis in the SM:

"*While the PRTF is an established and robust method to evaluate the resolution of conventional CDI reconstructions, its meaning is not straightforward when applied to Dichography. Indeed, the PRTF cannot disentangle the two contributions from the two frames, and consequently it is not possible to directly assign an individual resolution to the two reconstructed images. Furthermore, the resolution cannot be uniquely defined. Indeed, due to the different photon energies, the recorded signal at a specific scattering angle corresponds to two different momentum transfers, and thus to two different spatial resolutions depending on the X-ray color.*"

5. It is not well-described what the initial conditions are used for in Dichography. In standard phase retrieval algorithms, it is common to begin with a random initial phase to initiate the reconstruction. In Dichography, however, not only the object phases but also the modulation term ϕ^M must be retrieved. This implies that ϕ^M requires an initial setting as well. Do the authors initialize ϕ^M with a uniform value (e.g., 1 at all points), or do the authors start from a random distribution? Please clarify what initial condition was used and the rationale behind this choice

This aspect, while rather technical, is indeed a crucial step for phase retrieval algorithms. We have added a new paragraph at the end of the section "Adaptation of Memetic Phase Retrieval" in the main manuscript to explicitly detail these initial conditions and their significance.

As mentioned in the manuscript, Dichography utilizes the SPRING memetic framework (Colombo et al, 2025). A key feature of this framework is that it does not initialize reconstructions with random phases in Fourier space. Instead, it performs initialization in direct space, which provides several advantages for stability and convergence.

Each reconstruction in our parallel "population" begins with a direct-space matrix filled with several spherical density profiles of random positions and sizes (as described in the SPRING paper). This approach ensures that the initial Fourier phases already possess spatial correlations, preventing the first iterations of the algorithm from being overly "destructive". Furthermore, it allows for an initial normalization of the densities to match the L2 norm of the experimental data, which is otherwise impossible due to the missing central peak (beam stop).

In the context of Dichography, we must also initialize the relative weight between the two frames (corresponding to the modulation term phi^M mentioned by the referee). Each reconstruction is assigned a random initial "contribution balance." This ratio ranges from 50-50 (equal contribution) to 10-90 (one frame is nine times brighter than the other). Both initial densities are then globally scaled so that their sum matches the L2 norm of the experimental diffraction pattern. This is to ensure the "population" of reconstructions explores a wide range of the error landscape. Importantly, the algorithm is not constrained by this initial guess; a reconstruction starting with a 50-50 ratio can freely converge to a highly imbalanced solution if that provides a better fit to the

data. This stochastic initialization makes the process more robust and removes the need for case-by-case manual optimization of the intensity ratio.

6. In the last paragraph on page 4 of the Supplemental Material, it is stated that small samples are more resilient to noise because of their higher oversampling degree. However, smaller samples usually produce weaker scattering signals, which lowers the signal-to-noise ratio (SNR) and typically makes successful reconstruction more challenging. By contrast, larger samples scatter more strongly because of their larger cross-sections, resulting in higher SNR. For this reason, the current explanation appears inconsistent with the expected physical behavior. We suggest revising this paragraph to address this discrepancy.

Indeed, for a constant field intensity in the focus, the scattering cross-section scales with the volume of the sample (D^3), while the number of unknown pixels to retrieve scales with the projected area (D^2). Consequently, the ratio of recorded information to unknowns actually improves as the sample size increases, which would theoretically favor larger samples.
However, a second and often more restrictive factor in XFEL imaging is the central beam stop or hole in the detector which results in missing information at low q values. Larger samples produce closer diffraction orders in reciprocal space, and, consequently, as the sample size increases, more of the crucial low-frequency information is lost in the detector's central hole. This missing information creates a practical upper limit on the sample size that can be reconstructed, which is often much more impactful than the theoretical oversampling limit. In the revised Supplemental Material (Section S1.1), we have rewritten the respective paragraph to better reflect these competing factors. We now explain that while larger samples offer better SNR, they are simultaneously more vulnerable to the loss of low-frequency data. The "resilience" of smaller samples is therefore not just a matter of oversampling, but of ensuring that a sufficient number of diffraction orders are recorded outside the central blind spot of the detector.

Reviewer #3:

I co-reviewed this manuscript with one of the reviewers who provided the listed reports. This is part of the Nature Communications initiative to facilitate training in peer review and to provide appropriate recognition for Early Career Researchers who co-review manuscripts.

We also thank Reviewer #3 for their valuable work.
